# Whole-Genome Resequencing Identifies Loci Associated with Body Weight and Body-Size-Related Head Height in Bighead Carp (*Hypophthalmichthys nobilis*)

**DOI:** 10.3390/biology15201896

**Published:** 2026-10-21

**Authors:** Geng Chen, Fangyuan Peng, Dan Wang, Jingou Tong, Jin Xu

**Affiliations:** 1 Yangtze River Fisheries Research Institute, Chinese Academy of Fishery Sciences, Wuhan 430223, China; chengeng@yfi.ac.cn (G.C.);; 2 Institute of Hydrobiology, Chinese Academy of Sciences, Wuhan 430072, China

**Keywords:** bighead carp, whole-genome resequencing, genome-wide association study, body weight, head height, linkage disequilibrium, candidate genes

## Abstract

Growth traits are economically important in bighead carp (*Hypophthalmichthys nobilis*), but their genetic basis remains unclear. We conducted whole-genome association analyses in 298 fish using 2,891,492 single-nucleotide polymorphisms and linear mixed models controlling for population structure and genomic relatedness. Five variants reached the per-trait genome-wide threshold. The strongest body-weight association occurred at 13:40990738:C:T on linkage group 13 (LG13) (*p* = 5.09 × 10^−10^; effect = 5.50 ± 0.87 g). Two LG04 variants were associated with body weight, while two adjacent LG14 variants were associated with head height (lead *p* = 8.34 × 10^−9^; effect = 2.59 ± 0.44 mm). However, associations with head dimensions were substantially attenuated after body-length adjustment, indicating that head-specific genetic effects were not established. Regional linkage disequilibrium and conditional analyses distinguished compact LG13 and LG14 signals from a complex LG04 pattern, and complementary sensitivity analyses supported their robustness. Sanger sequencing confirmed all 118 tested LG13 genotypes. The LG13 body-weight association was reproduced in an independent population of 268 fish and remained significant after body-length adjustment. Genotype-stratified reverse-transcription quantitative polymerase chain reaction assays detected higher expression of *mchr2*-like gene13.20, *syngr1*-like gene32.45, and *cacng2*-like gene32.46 in effect-allele carriers after multiple-test correction. These findings provide independent support for the LG13 body-weight association, while LG14 and LG04 remain discovery-stage candidate regions requiring independent replication and functional investigation before potential breeding applications can be considered.

## Simple Summary

Bighead carp is an important freshwater aquaculture fish valued for its rapid growth and large edible head, yet the genetic basis of these traits remains poorly understood. We examined approximately 2.89 million genetic variants across the genome in 298 fish and identified three major regions related to body weight or head height. The strongest body-weight signal was accompanied by additional body-weight and head-height signals in two other regions. Several complementary analyses showed that these associations remained stable under different statistical assumptions and data-quality filters. The key genetic variant associated with body weight was confirmed using a second testing method in the original fish and was also associated with greater body weight in another 268 fish derived from different parents and raised in a different pond. Measurements of gene activity further supported three candidate genes near two of the associated regions. Together, these findings identify a body-weight-associated marker supported by an independent population and additional preliminary genomic regions associated with growth-related traits. However, the head-height associations mainly reflected overall body-size variation, and further validation is needed before these markers can be considered for selective breeding.

## 1. Introduction

Bighead carp (*Hypophthalmichthys nobilis*) is an important freshwater aquaculture species for which both body weight and head-related characteristics are relevant to production and product evaluation [1,2,3]. The head is valued as an edible portion, particularly in Chinese culinary applications. However, quantitative information on regional market prices, consumer preferences, and processing yields was not collected in the present study. Therefore, we do not assume that either larger absolute head dimensions or a greater head-to-body proportion necessarily confers a higher economic return. In this study, body weight and absolute head dimensions were examined as primary growth-related traits, whereas the head-to-body length ratio and body-length-adjusted analyses provided complementary assessments of relative head morphology. Chromosome-level reference assemblies and sequence-based genotyping now support detailed investigation of these quantitative traits, while genomic approaches are increasingly being incorporated into aquaculture breeding [1,4,5].

Previous studies have established a genetic basis for growth and head-related variation in bighead carp. Fu et al. identified growth-related quantitative trait loci (QTL) using a high-density linkage map [6], and Liu et al. refined growth-related intervals and proposed *tp53bp2* as a candidate gene [7]. A type IIB restriction site-associated DNA (2b-RAD) genome-wide association study (GWAS) of 776 fish mapped head-size and head-shape associations [2]. A subsequent analysis of growth and body conformation in 776 individuals highlighted LG16 and LG21 and provided additional evidence for *fndc5b* [3]. Pedigree and bulked-segregant analyses further supported a quantitative genetic basis for growth traits [8]. Although previous 2b-RAD-based GWAS identified genomic regions associated with head dimensions, growth, and body conformation in bighead carp, the local linkage disequilibrium (LD) structure and conditional dependence of association signals remain incompletely characterized [2,3]. Furthermore, distinguishing genetic associations with absolute head dimensions from those reflecting overall body size remains important for interpreting head-related traits. To address these questions, the present study used approximately 2.89 million single-nucleotide polymorphisms (SNPs) derived from whole-genome resequencing of an additional cohort to characterize regional LD, perform conditional association analyses, and examine body-length-adjusted head phenotypes. We further assessed the strongest body-weight-associated marker through orthogonal genotyping and evaluation in an independent population. These analyses complement, rather than replace, previous studies based on larger cohorts and reduced-representation genotyping.

Head dimensions can be studied as absolute measurements or relative to overall body size, and these definitions answer different biological and breeding questions. Common carp studies have analyzed both head length and the head-to-body length ratio [9], whereas body-shape GWAS in large yellow croaker have used several proportional traits [10]. Size-adjusted head phenotypes have also been studied in catfish [11]. Thus, body-size adjustment is useful when the objective is relative morphology, but it is not a prerequisite for mapping absolute head dimensions. Explicit log-scale adjustment can help characterize the relationship between these trait definitions [12,13]. Here, absolute size measurements are the main association targets, with the head-to-body length ratio and body-length-adjusted models providing complementary information.

Dense association mapping can also help connect a regional signal to candidate genes. LD and conditional tests characterize which marker associations remain after accounting for representative variants, while protein similarity and expression data help prioritize genes for follow-up [14]. In bighead carp, transcriptomic analysis of the hypothalamus–pituitary–liver axis has linked growth differences to neuroendocrine and metabolic processes [15]. Integrating such public data with GWAS is useful for biological interpretation, although expression in independent fish does not itself validate a marker–gene relationship.

We analyzed whole-genome resequencing data from 298 juvenile bighead carp reared in a common pond to map associations with body weight, linear dimensions, and head-to-body length ratio. We characterized the principal regions using local LD, conditional association tests, and genotype–phenotype summaries; examined sensitivity to population structure, phenotype scale, and genotype quality; and prioritized nearby genes using reference annotation and public RNA sequencing (RNA-seq) data. We then retyped the strongest marker in discovery fish, tested it in an independent cohort, and evaluated four regional candidates using genotype-stratified reverse-transcription quantitative polymerase chain reaction (RT-qPCR). The aim was to define and independently assess genetic targets for commercially relevant size traits.

## 2. Materials and Methods

### 2.1. Experimental Fish and Phenotypic Measurements

Fish hatched in May 2025 and were reared together in one pond at the Liangzi Lake Experimental Base of the Yangtze River Fisheries Research Institute, Chinese Academy of Fishery Sciences, Wuhan, China, until sampling in February 2026, at approximately nine months of age. According to hatchery records, ten female and seven male broodfish were used for natural spawning. However, the identities and number of broodfish that actually contributed offspring were not determined, and individual parentage could not be assigned. During the summer-fingerling nursery stage, the stocking density was approximately 150,000 fish/ha. In August 2025, the stocking density was adjusted to approximately 30,000 fish/ha. As a filter-feeding species, bighead carp relied primarily on naturally occurring plankton in the pond, and no supplementary formulated feed was provided throughout the rearing period. Mortality was not systematically recorded during cultivation, and the overall mortality rate could therefore not be calculated. Daytime records from May to September 2025, the main culture season, showed water temperatures of 22–30 °C and dissolved oxygen concentrations of 4.8–6.1 mg/L. Sex was not determined because the juvenile fish were sampled nonlethally, without gonadal examination or molecular sex determination. Individual pedigree information was unavailable.

After fasting for 3 d, 310 fish were anesthetized with tricaine methanesulfonate (MS-222; Gaiyuntian, Wuhan, China) and phenotyped. Body weight (BW) was recorded to 0.1 g using a balance (Laike, Beijing, China); total length (TL), body length (BL), head length (HL), and head height (HH) were measured with a digital caliper (Jinnong, Weifang, China) with 0.01 mm resolution. BL extended from the snout tip to the caudal-fin base, and HL from the snout tip to the posterior margin of the opercular bone. HH was the vertical distance from the highest dorsal point of the head to the ventral surface directly below it. Head-to-body length ratio (HBR) was calculated as HL/BL. No observation was excluded solely for an extreme phenotype. Caudal-fin samples were preserved in 95% ethanol; fish were not euthanized for fin sampling. Procedures were approved by the institutional Animal Ethics Committee (YFI2026110).

### 2.2. DNA Sequencing, Alignment, and Variant Processing

Genomic DNA was extracted by the phenol–chloroform method and assessed using NanoDrop spectrophotometry and Qubit fluorometry (Thermo Fisher Scientific, Waltham, MA, USA), together with 1% agarose-gel electrophoresis (Biosharp, Hefei, China). Acceptance criteria were OD260/280 = 1.8–2.0, OD260/230 ≥ 2.0, concentration ≥ 5 ng/μL, total DNA ≥ 0.2 μg, and no obvious degradation. Twelve samples failed DNA quality control and were not used for library preparation. Annoroad Gene Technology Co., Ltd. (Beijing, China) prepared libraries with approximately 350 bp inserts from the remaining 298 fish and sequenced 150 bp paired-end reads on a DNBSEQ-T7 platform (MGI Tech Co., Ltd., Shenzhen, China), targeting approximately 10× depth per individual. The actual mean sequencing depth across the 298 individuals, calculated using SAMtools depth, was 12.93 ± 1.42× (mean ± standard deviation [SD]), with a median of 13.02× (interquartile range, 11.81–14.09×) and a range of 9.92–15.83×.

Reads were processed with fastp v0.24.0 using automatic paired-end adapter detection, front and tail trimming with a 4 bp sliding window and mean quality threshold of 20, and a minimum retained length of 50 bp [16]. Clean reads were aligned to the genome of Fu et al. [1] using BWA-MEM2 v2.2.1 with sample-specific read groups [17]. Alignments were coordinate sorted with SAMtools v1.21 [18], and duplicates were marked with Sambamba v1.0.1 [19]. HaplotypeCaller in GATK v4.6.2.0 generated per-sample genomic variant call format (gVCF) files, which were imported by reference sequence with GenomicsDBImport and jointly genotyped with GenotypeGVCFs [20].

BCFtools v1.21 was used to concatenate VCFs and retain biallelic SNPs [18]. Site-level filtering excluded QUAL < 30 or INFO/DP < 3, retaining 10,581,717 of 10,582,828 biallelic SNPs. Subsequent filtering with VCFtools v0.1.16 required a minimum site call rate of 80% (--max-missing 0.8) and a minor allele frequency (MAF) of at least 0.05 (--maf 0.05), yielding 3,866,041 SNPs. After excluding 3003 SNPs on unplaced contigs, 3,863,038 SNPs on LG01–LG24 remained and were converted to binary format using PLINK v1.90b6.21 [21], without additional variant filtering. No Hardy–Weinberg equilibrium filter or additional per-genotype depth or genotype-quality threshold was applied during this primary SNP-filtering stage. Genotype-quality masking was evaluated separately in the subsequent LG13 sensitivity analyses. The MAF threshold was applied to reduce unstable association estimates arising from low minor-allele counts in the 298-fish discovery population. The overall genotyping rate was 96.22%, and maximum individual missingness was 8.09%. GEMMA applied a stricter site-level genotype missingness threshold of 5%, excluding 969,189 SNPs with missingness exceeding this threshold. An additional 2357 SNPs were excluded because their nonmissing genotype dosages showed no variation, leaving 2,891,492 SNPs for association analysis.

### 2.3. Population Structure and Primary Association Models

SNPs were LD-pruned in PLINK using a 50-SNP window, a five-SNP step, and *r*^2^ = 0.2. Principal component analysis (PCA) was conducted using the resulting 532,418 SNPs. The proportion of genetic variation explained by each PC was calculated by dividing its eigenvalue by the sum of all eigenvalues. Exploratory k-means clustering used unscaled PC1–PC2 scores with four clusters, 100 random starts, and seed 2026; these clusters were used for descriptive and sensitivity analyses. Individual genotype-missingness proportions were summarized by the four inferred genetic clusters. Within C3, missingness was also summarized separately for TT homozygotes and C-allele carriers at the LG13 lead SNP. Individuals without a callable LG13 lead genotype were tabulated separately. These comparisons were descriptive and were not intended to establish whether missingness was random at individual genomic loci. Overall and pairwise genetic differentiation among clusters was quantified using Weir–Cockerham fixation index (FST) estimates calculated in PLINK from the same LD-pruned SNP set, with weighted mean FST values reported as the primary estimates. Differences in BW among clusters were assessed using Welch’s one-way analysis of variance (ANOVA), followed by pairwise Welch’s *t*-tests with Holm adjustment for multiple comparisons. The Kruskal–Wallis test was additionally used as a nonparametric sensitivity analysis. A centered genomic relatedness matrix was calculated in GEMMA v0.98.5 [22].

Each of the six primary traits (BW, TL, BL, HL, HH, and HBR) was tested using a univariate GEMMA linear mixed model with an intercept, PC1–PC5, SNP allele dosage, and a random genetic effect defined by the relatedness matrix. The first five PCs were selected to account for the major axes of population stratification, based on inspection of the eigenvalue distribution of the first 20 components (Figure S1). The PCs were included as fixed-effect covariates, whereas the genomic relatedness matrix modeled pairwise genetic relatedness as a random effect. HBR was standardized to zero mean and unit sample standard deviation for numerical stability. Primary inference used likelihood-ratio-test (LRT) *p* values (*p*_LRT_). Per-trait genome-wide and suggestive thresholds were 0.05/N = 1.729 × 10^−8^ and 1/N = 3.458 × 10^−7^, respectively, with N = 2,891,492. The effective number of independent trait tests was estimated as 3.0 using the eigenvalue-based method of Li and Ji [23], applied to the Pearson correlation matrix of the six primary traits. Given the linkage disequilibrium among SNPs, the Bonferroni correction based on all tested markers was considered conservative. The reported thresholds control marker multiplicity within each trait, rather than across all six traits; the effective number of independent trait tests was reported for context and was not used to redefine these thresholds. Non-finite *p* values were treated as unavailable without reducing N. Genomic inflation (λGC) was calculated from the median one-degree-of-freedom chi-square statistic relative to its null median.

The genomic proportion of variance explained (PVE) and its standard error were estimated from the GEMMA null models for the six primary traits, with an additional estimate obtained for log-transformed BW. These estimates represent the proportion of phenotypic variance attributed to the genomic-relatedness random effect under the fitted model and were interpreted cautiously because of their substantial standard errors. Pairwise genetic correlations among the six primary traits were estimated using bivariate GEMMA mixed models incorporating PC1–PC5 and the genomic relatedness matrix. Genetic correlations were calculated from the estimated genetic covariance and variance components of each bivariate model. Because the trait pairs were fitted separately, the resulting correlation estimates were not constrained to form a positive-semidefinite joint correlation matrix. Estimates approaching the parameter boundaries were therefore considered exploratory.

### 2.4. Regional LD and Conditional Association Analysis

Regional analyses focused on 13:40990738:C:T, 14:13799852:C:A, and three representative SNPs on LG04: 4:30566806:A:T, 4:32624853:G:T, and 4:33112675:C:T. The LG04 SNPs were selected to represent spatially separated local association peaks, including two genome-wide significant BW signals (4:32624853 and 4:33112675) and one suggestive HL signal (4:30566806), which was also suggestively associated with BW. PLINK pairwise *r*^2^ values described local LD; lead-centered high-LD intervals spanned markers with *r*^2^ ≥ 0.8 to the lead. Conditional models added the corresponding lead dosages to the primary covariates while retaining the same relatedness matrix. Missing conditioning dosages were mean-imputed. Tests included individual and joint conditioning on the LG04 representatives and cross-region conditioning for BW and HH. Regional summaries used LG13:40,840,000–41,130,000, LG14:13,650,000–13,950,000, and LG04:30,450,000–33,250,000 bp. Conditioning markers were excluded from residual regional summaries. Raw genotype-group means were descriptive; rare homozygotes were combined with heterozygotes for selected carrier plots, without inferring an inheritance mechanism.

To quantify local statistical support for the representative association signals, neighboring SNPs were examined within ±10, ±50, and ±100 kb of each lead variant using the original GEMMA association results. Four representative variants were evaluated for BW (LG13 and LG04-A, -B, and -C), and the LG14 lead was evaluated for HH. The lead SNP itself was excluded from neighboring-SNP counts. Only neighboring SNPs with finite positive likelihood-ratio-test *p* values were included, and counts were summarized at *p* < 1 × 10^−5^, *p* < 1 × 10^−6^, the per-trait suggestive threshold (*p* ≤ 1/N), and the genome-wide significance threshold (*p* ≤ 0.05/N), where N = 2,891,492. The ±50 kb window was used for the primary regional summary, with the other windows providing additional context.

Bayesian fine-mapping was performed using SuSiE for the LG13–BW (40.84–41.13 Mb), LG14–HH (13.65–13.95 Mb), and LG04–BW (30.45–33.25 Mb) regions. Genotypes were extracted from the discovery cohort and restricted to SNPs included in the corresponding primary GEMMA analyses, yielding 1941, 1021, and 10,995 SNPs, respectively, in 298 individuals. Missing genotype dosages were imputed using the corresponding SNP-specific mean dosages. To approximately account for population structure and relatedness, phenotypes and genotype matrices were whitened using the covariance matrix V = vgK + veI, where K was the original genomic relatedness matrix and vg and ve were the genetic and residual variance components estimated from the corresponding GEMMA null models. The intercept and PC1–PC5 were subsequently removed by orthogonal projection. SuSiE was fitted to the transformed individual-level data with a maximum of five single effects, standardized genotype predictors, fixed residual variance of one, and estimated prior effect variances. Posterior inclusion probabilities (PIPs) and nominal 95% credible sets were calculated. Credible sets were evaluated using a minimum absolute within-set correlation threshold of 0.5, and sets failing this purity criterion were reported separately as unfiltered, low-purity sets. Fine-mapping results were considered exploratory and conditional on the specified model, prior assumptions, and analyzed variants.

The robustness of the LG04 conditional associations was assessed by excluding individuals carrying the rare AA homozygous genotype at LG04-A (*n* = 3), the rare CC homozygous genotype at LG04-C (*n* = 3), or both. One individual belonged to both rare-homozygote groups; thus, the combined exclusion involved five unique individuals. The resulting sample sizes were 295, 295, and 293, respectively, compared with 298 in the full cohort. For each exclusion scenario, the BW association of each LG04 representative SNP was retested using a GEMMA linear mixed model, with the other two representative SNPs included as conditioning covariates. The original PC1–PC5 covariates and genomic relatedness matrix were retained, and missing conditioning genotypes were mean-imputed. Model sensitivity was evaluated by comparing effect directions, effect magnitudes, standard errors, and likelihood-ratio-test *p* values with those obtained from the full cohort.

### 2.5. Sensitivity and Body-Length-Adjusted Analyses

Natural-log-transformed BW was tested using the primary model. Sensitivity analyses of the LG13 lead SNP included three approaches. First, leave-one-chromosome-out (LOCO) models used a relatedness matrix excluding LG13 to reduce proximal contamination. These models compared PC1–PC5 adjustment alone with additional cluster indicators. C3 was selected as the reference category because all LG13 C-allele carriers belonged to this cluster; this choice only determined the coding of the cluster covariates. Second, the association was examined within C3 using C3-specific PCs and an LG13-excluded relatedness matrix to reduce confounding by between-cluster differences. Third, genotype-quality sensitivity tests masked calls with read depth (DP) < 4 or genotype quality (GQ) < 10, or with the stricter DP < 5 or GQ < 20 rule, using the +setGT plugin in BCFtools v1.21. The corresponding site-missingness limits were 0.10 and 0.30, respectively, to retain the LG13 lead SNP, whose missingness increased to 7.72% and 24.83% after masking. These site-missingness limits were applied only to the targeted LG13 genotype-quality sensitivity analyses and did not alter the SNP-filtering criteria used in the primary genome-wide association analysis. These tests retained PC and cluster covariates and the LOCO matrix. All sensitivity analyses assessed the robustness of the previously identified association and did not constitute independent replication.

To further characterize potential confounding by genetic cluster membership, descriptive BW comparisons were performed between C3 and the pooled C1, C2, and C4 clusters, both in the full cohort and after restricting the analysis to TT homozygotes at the LG13 lead SNP. Mean BW was also summarized separately for TT individuals in each cluster. Within C3, CT and CC individuals were combined as C-allele carriers and compared with TT homozygotes. Two-sided Welch’s *t*-tests were used to evaluate these differences, with mean differences, 95% confidence intervals, and *p* values reported. These unadjusted genotype-stratified comparisons were considered descriptive and were interpreted separately from the PC- and relatedness-adjusted LOCO mixed-model analyses.

To compare the variance explained by the LG13 lead SNP between cohorts, single-marker ordinary least-squares (OLS) regressions of BW and ln(BW) on C-allele dosage were fitted separately in the discovery and independent cohorts. These analyses were restricted to individuals with nonmissing genotypes, and the resulting sample R^2^ values were interpreted as descriptive proportions of observed phenotypic variance explained within each cohort, rather than estimates of genetic variance explained or out-of-sample predictive accuracy. In the discovery cohort, partial R^2^ values were additionally calculated for the LG13 SNP after adjustment for PC1–PC5, with and without cluster indicators, as the reduction in residual sum of squares relative to the corresponding covariate-only OLS model. The independent-cohort regressions were also repeated after excluding the three CC homozygotes. Potential discovery-stage effect inflation was considered by comparing absolute and log-scale effect estimates between cohorts, while recognizing that these descriptive comparisons do not provide a formal correction for winner’s curse.

As secondary analyses of head dimensions relative to body length, ln(HL) and ln(HH) were tested in mixed models additionally including ln(BL), with the same PCs and relatedness matrix. Outcomes were standardized for numerical stability; reported effects and standard errors were back-transformed to the natural-log scale. Descriptive ordinary least-squares regressions summarized log–log scaling, with slope = 1 tested against a two-sided alternative. Their residuals were used for visualization. In the initial body-length-adjusted HL analysis, 7338 SNPs returned non-finite likelihood-ratio-test *p* values. These SNPs were retested using an alternative response defined as the OLS residual from ln(HL) regressed on ln(BL), divided by its sample standard deviation. The retry models retained the intercept, ln(BL), PC1–PC5, and the same genomic relatedness matrix. Because this transformation subtracts a linear combination of covariates already included in the mixed model and rescales the response, the likelihood-ratio tests are theoretically invariant under successful model fitting. Numerical consistency was verified for 264 control SNPs distributed across all 24 linkage groups, with a maximum absolute difference of 8.71 × 10^−8^ in −log10(*p*) between the original and transformed-response models. The retry procedure recovered finite *p* values for 7279 SNPs. The remaining 59 SNPs (0.0020% of all tested SNPs) were distributed across 22 linkage groups, with a maximum of three unresolved SNPs in any 1 Mb genomic window, indicating no pronounced regional concentration. These 59 *p* values were treated as unavailable rather than assigned artificial numerical values. Valid initial *p* values were preserved, and the total number of tested SNPs used for multiple-testing correction was unchanged. These conditional analyses addressed a different phenotype definition from the primary absolute-dimension GWAS.

To further assess whether associations with absolute head dimensions persisted after accounting for overall body weight, the five representative lead SNPs were reanalyzed for HL and HH using univariate mixed models with BW included as an additional fixed-effect covariate. These models retained PC1–PC5 and the same genomic relatedness matrix as the corresponding primary analyses. The BW-adjusted tests were considered secondary sensitivity analyses rather than independent tests of direct genetic effects.

### 2.6. Candidate-Gene Annotation

Regional genes were manually prioritized using genomic proximity, LD context, predicted protein features, and BLASTP sequence similarity [24], using the genome and annotation of Fu et al. [1]. Distances were measured to the nearest gene boundary. Full annotation identifiers and coordinates were retained for matching to expression results. Protein similarity searches were performed using BLASTP through the National Center for Biotechnology Information (NCBI) web interface (https://blast.ncbi.nlm.nih.gov/ accessded on 17 October 2026) rather than a locally installed executable. The LG13 gene82.0 model was rechecked against the ClusteredNR database. Short or weakly supported models were left unresolved or assigned tentative annotations. The selected genes constitute a prioritized regional set rather than an exhaustive list of possible causal genes.

The approximately 8 kb lead-centered high-LD interval on LG13 was further subjected to sequence-based annotation. The interval was defined by the outermost SNP positions with pairwise *r*^2^ ≥ 0.8 relative to the lead variant (LG13:40,990,738–40,998,720; 7983 bp, inclusive). The corresponding reference sequence was extracted from the bighead carp genome, and overlaps with features annotated in General Feature Format version 3 (GFF3) were examined. Low-complexity regions were identified using dustmasker v1.0.0, and tandem repeats were screened using Tandem Repeats Finder (TRF) v4.09 (parameters: 2 7 7 80 10 50 500). Interspersed repetitive elements were annotated using RepeatMasker v4.2.3 with RMBlast v2.17.1+ and Dfam v3.9, specifying Cyprinidae and including both curated and uncurated repeat families. RepeatMasker output was examined to determine repeat classes, sequence coverage, and the overlap of the six high-LD SNPs with annotated repeat intervals. These computational annotations were interpreted as sequence-based predictions rather than evidence of experimentally established regulatory activity.

### 2.7. Public RNA-Seq Analysis and Statistical Summaries

Public RNA-seq data comprised 27 libraries from PRJNA1253371, PRJNA721309, PRJNA1303128, and PRJNA453809, independent of the GWAS fish. Reads were aligned to the same reference with STAR v2.7.11b [25] and counted with featureCounts v2.0.6 [26]. Descriptive profiles used 15 libraries: three High-group muscle libraries (PRJNA1253371), three Normal_0h liver and three Normal_0h gill libraries (PRJNA721309), three Pond_reared intestine libraries (PRJNA1303128), and three High-group mixed hypothalamus–pituitary (HP) libraries (PRJNA453809) [15]. Transcripts per million (TPM) were calculated per library from counts and featureCounts gene lengths; arithmetic means across the three libraries per group were displayed as log2(mean TPM + 1). These are tissue–condition profiles, not a controlled comparison of tissue-specific expression.

High-versus-Low differential expression was tested separately within tissue and BioProject: HP and liver from PRJNA453809, and muscle from PRJNA1253371, each with three libraries per group. Genes with ≥10 reads in at least three libraries were retained. DESeq2 v1.42.1 used design ~ Group, with Low as reference, Wald tests, and Benjamini–Hochberg (BH) adjustment within each comparison [27]. Differential expression required adjusted *p* < 0.05 and absolute apeglm-shrunken log2 fold change ≥ 1 (apeglm v1.24.0) [28]. Candidate-level fold changes reported here are unshrunken estimates. Full gene IDs were used throughout, and prefiltered or otherwise unavailable results were distinguished from non-significant tests. Phenotypic summaries, Pearson correlations, and visualizations were prepared in R v4.4.0 for the 298 GWAS fish. All GWAS *p* values reported below are LRT values; suggestive-only counts exclude genome-wide significant SNPs.

### 2.8. Orthogonal Genotyping and Independent-Cohort Validation

The LG13 lead SNP was retyped by PCR and Sanger sequencing using primer pair P1 (forward, 5′-CCATAGATGGTCATGTTCTTC-3′; reverse, 5′-CTCAAGAGCTATAAGCCTGG-3′; expected amplicon, 424 bp). The 118 discovery-cohort fish comprised all 62 calls newly masked by the stringent DP < 5 or GQ < 20 rule, the remaining 26 high-quality C-allele carriers, and 30 high-quality TT controls. Fish lacking an original callable whole-genome-sequencing genotype at this site were not included in the targeted concordance set.

Independent replication used 268 bighead carp hatched in May 2022 and sampled in May 2023 at approximately 12 months of age from a different pond at the Zhangdu Lake Experimental Base, Wuhan, China. Their parents differed from those of the discovery cohort. Fish were sampled without reference to phenotype in measurement order and assigned sequential identifiers. BW, BL, HL, and HH were recorded, and the LG13 lead was genotyped with the same amplicon-based assay. Hardy–Weinberg equilibrium was evaluated by an exact test. The primary replication analysis regressed BW on C-allele dosage (0, 1, or 2) by ordinary least squares; ln(BW) and the linear traits were secondary outcomes. Models additionally including BL or ln(BL) assessed whether the BW association persisted conditional on body length. Given the unequal genotype-group sizes and the presence of only three CC homozygotes, robustness was assessed using HC3 heteroskedasticity-consistent standard errors and by repeating the BW analyses after excluding CC fish. The available breeding records did not permit determination of the number of contributing parental pairs or assignment of individuals to families. Therefore, family effects could not be included in the replication models. Because only the target LG13 locus was genotyped, genome-wide PCs and a genomic relatedness matrix could not be estimated. Consequently, the single-marker OLS analyses could not explicitly account for pedigree relatedness, and their statistical significance should be interpreted with this limitation in mind.

### 2.9. Genotype-Stratified RT-qPCR Analysis

The fish used for targeted RT-qPCR were obtained from the same 2025 breeding cohort as the GWAS discovery population but were sampled at a later time point. Specifically, 15 individually identified bighead carp (T1–T15) were sampled in June 2026 from the bighead carp culture pond at the Liangzi Lake Experimental Base, whereas the GWAS discovery population had been sampled in February 2026. These RT-qPCR samples were separate from the 268-fish independent validation cohort used for the LG13 body-weight association analysis. Genotypes at the representative SNPs on LG13 (13:40990738:C:T), LG14 (14:13799852:C:A), and LG04 (4:32624853:G:T) were determined by locus-specific PCR amplification followed by Sanger sequencing. For each candidate gene, five reference-homozygous fish and five effect-allele carriers were selected for expression comparison. Specifically, gene82.0 was compared between TT fish and C-allele carriers at LG13, gene13.20 between AA fish and C-allele carriers at LG14, and gene32.45 and gene32.46 between TT fish and G-allele carriers at LG04. The same ten fish were used for the gene32.45 and gene32.46 comparisons, whereas the selected sets for the other genes partially overlapped. Individual BW records were matched to the selected fish and summarized by genotype group. Intestinal tissue was analyzed for gene82.0, whereas mixed hypothalamus–pituitary tissue was analyzed for gene13.20, gene32.45, and gene32.46. These genotype-stratified expression comparisons were not considered independent marker–phenotype replication.

Total RNA was extracted with TRIzol reagent (Invitrogen, Thermo Fisher Scientific, Waltham, MA, USA), and complementary DNA (cDNA) was synthesized using the PrimeScript RT Reagent Kit (TaKaRa, Kusatsu, Japan). Quantitative PCR was performed with TB Green^®^ Premix Ex Taq™ (TaKaRa) on a QuantStudio 1 Plus Real-Time PCR System (Applied Biosystems, Thermo Fisher Scientific, Waltham, MA, USA). *β-actin* was used as the reference gene (forward primer, 5′-ACAGGCTCCTATTCAATGTTTCC-3′; reverse primer, 5′-CGCCCATCCAGAAGGTGTAA-3′; amplicon, 131 bp). Each 10 μL reaction contained 5 μL of 2× TB Green^®^ Premix Ex Taq™, 0.2 μL each of the forward and reverse primers, 1 μL of cDNA template, and 3.6 μL of nuclease-free water. Thermal cycling conditions began with 30 s incubation at 95 °C, then 40 cycles of 5 s at 95 °C and 34 s at 60 °C. Each biological sample was analyzed in three technical replicates, and their mean quantification cycle (Cq) value was used for subsequent calculations. Previous work in bighead carp reported the use of *β-actin* as an RT-qPCR reference gene and evaluated reference-gene stability across tissues and developmental stages [29]. However, the suitability of a reference gene may depend on the tissues and experimental conditions being examined. In the present study, we performed an exploratory assessment of *β-actin* Cq consistency by comparing fish-level Cq values, averaged across three technical replicates, between the corresponding genotype groups for each of the four candidate-gene assays using two-sided Welch’s *t*-tests. No additional candidate reference genes were evaluated in the present hypothalamus–pituitary or intestinal samples; therefore, this assessment was not considered a formal multi-reference-gene stability validation. Amplification efficiencies were 96.7% for *β-actin*, 103.2% for gene82.0, 95.3% for gene13.20, 97.8% for gene32.45, and 92.2% for gene32.46. The corresponding standard-curve R^2^ values exceeded 0.98 for all assays. Melting-curve analysis showed a single peak for each primer pair, supporting amplification specificity.

Primer sequences and expected amplicon sizes for both Sanger genotyping and RT-qPCR assays are provided in Table S9. Relative expression was calculated by the 2^−ΔΔCt^ method [30]. Statistical tests used fish-level ΔCt values, with two-sided Welch’s *t*-tests for each carrier–reference comparison and Benjamini–Hochberg correction across the four prespecified genes; adjusted *p* < 0.05 was considered significant.

## 3. Results

### 3.1. Phenotypic Variation and Population Structure

**Figure 1:**
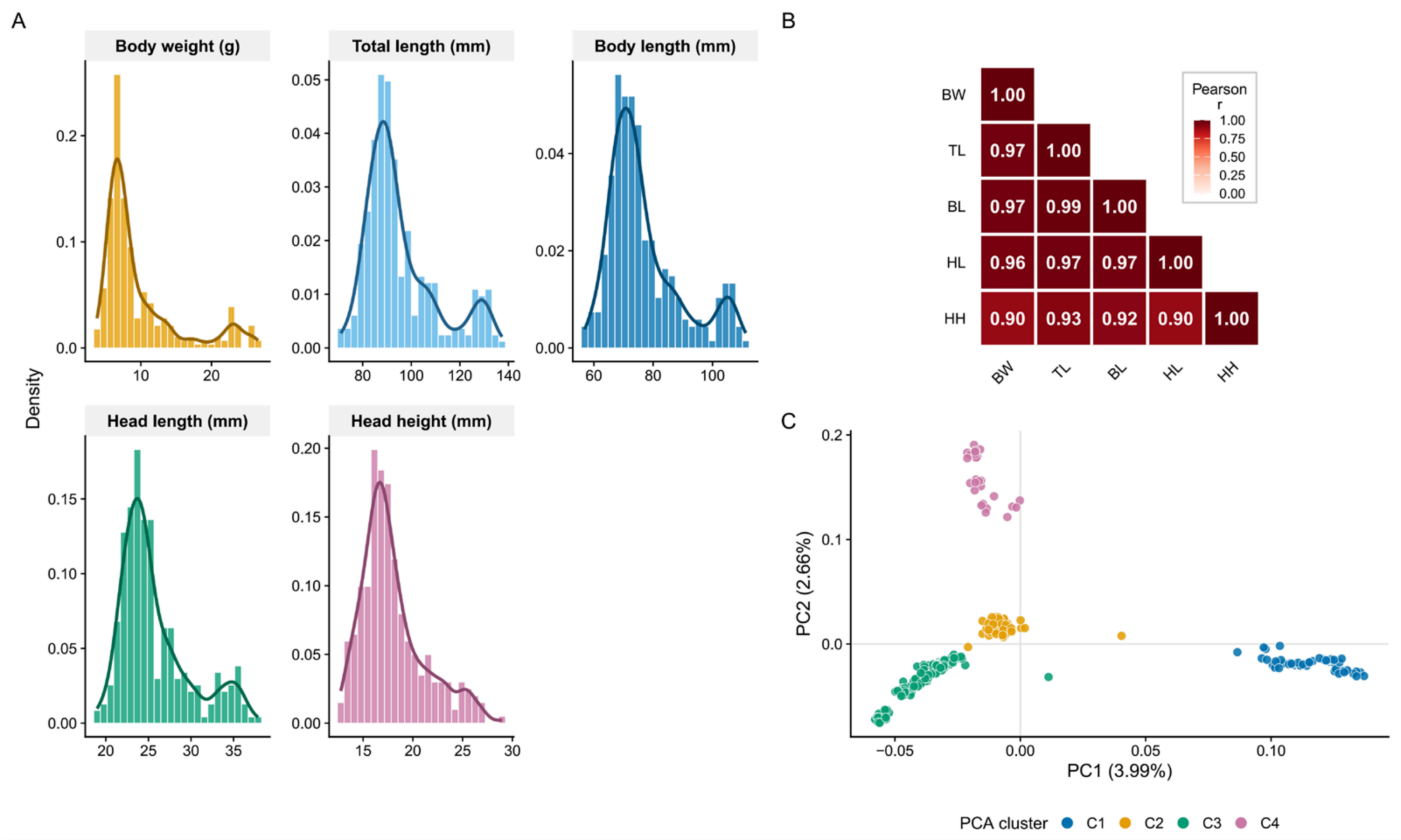
Phenotypic variation, correlations among growth-related traits, and genetic structure of the bighead carp discovery population. (**A**) Histograms and kernel density curves showing the distributions of body weight (BW), total length (TL), body length (BL), head length (HL), and head height (HH) in 298 genotyped individuals. Colors distinguish the five traits. (**B**) Lower-triangular Pearson correlation matrix for the five traits. Darker red indicates a stronger positive correlation, and the values within the cells represent Pearson’s correlation coefficients. (**C**) Principal component (PC) analysis based on 532,418 LD-pruned genome-wide SNPs. PC1 and PC2 explained 3.99% and 2.66% of the genetic variation, respectively. Each point represents one individual, and colors indicate the four exploratory clusters (C1–C4) identified by k-means clustering of PC1 and PC2.

A total of 298 individuals had both complete phenotype information and genome-wide genotype data. Considerable variation was observed for all absolute growth traits. Mean ± SD values were 9.68 ± 5.47 g for BW, 95.49 ± 14.87 mm for TL, 76.87 ± 12.36 mm for BL, 25.71 ± 4.08 mm for HL, and 17.96 ± 3.20 mm for HH (Figure 1; Table S1). BW showed the greatest relative variation, with a coefficient of variation (CV) of 56.47%. HBR averaged 0.3348 ± 0.0135 and had a much lower CV of 4.02%.

The five absolute size traits were strongly positively correlated (Figure 1; Table S1), with Pearson *r* ranging from 0.895 for BW–HH to 0.993 for TL–BL. Correlations of BW with TL, BL, HL, and HH were 0.974, 0.975, 0.962, and 0.895, respectively; HL–BL and HH–BL correlations were 0.968 and 0.924. HBR showed weaker correlations with absolute size traits (*r* = −0.169 to 0.080).

The genomic PVE estimates (± SE) were 0.119 ± 0.169 for BW, 0.024 ± 0.151 for TL, 0.011 ± 0.151 for BL, 0.139 ± 0.158 for HL, 0.041 ± 0.156 for HH, and 0.261 ± 0.146 for HBR (Table S2). Bivariate mixed models yielded positive pairwise genetic correlation estimates, including 0.929 between BW and HL and 0.998 between BW and HH. However, the PVE estimates had substantial standard errors, and several pairwise genetic correlations approached the upper parameter boundary. Because the genetic correlations were estimated separately for each trait pair, their combined matrix was not constrained to be positive semidefinite. These genetic parameter estimates were therefore interpreted as exploratory rather than precise measures of genetic architecture.

PCA based on 532,418 LD-pruned genome-wide SNPs revealed genetic heterogeneity among the 298 fish (Figure 1C). PC1–PC5 explained 3.99%, 2.66%, 1.66%, 1.47%, and 1.38% of the total genetic variation, respectively, accounting for 11.17% cumulatively. Exploratory k-means clustering of PC1 and PC2 assigned 55, 76, 138, and 29 individuals to C1, C2, C3, and C4, respectively. The overall weighted mean FST among the four clusters was 0.0491, with pairwise weighted FST values ranging from 0.0189 (C2–C3) to 0.1037 (C1–C4), indicating genetic differentiation among the exploratory clusters.

Mean individual genotype missingness was 3.64%, 3.96%, 3.79%, and 3.54% in clusters C1, C2, C3, and C4, respectively. Within C3, mean missingness was 3.69% among 105 TT homozygotes and 4.04% among 30 C-allele carriers (22 CT and eight CC individuals) at the LG13 lead SNP; three additional C3 individuals lacked a callable genotype at this locus. These descriptive results did not reveal pronounced differences in overall genotype missingness among clusters or between the LG13 genotype groups within C3. However, they do not establish that missingness was completely random with respect to genotype or genomic position.

Mean BW differed among clusters, with values of 7.29 ± 2.41 g for C1, 7.68 ± 2.69 g for C2, 11.97 ± 6.81 g for C3, and 8.53 ± 3.14 g for C4 (mean ± SD). The overall difference was significant (Welch’s ANOVA: F(3, 108.86) = 17.46, *p* = 2.51 × 10^−9^), consistent with the Kruskal–Wallis test (*p* = 2.55 × 10^−7^). Following Holm correction, C3 had significantly greater mean BW than C1, C2, and C4, whereas no significant differences were detected among C1, C2, and C4.

Although the fish were produced in the same breeding batch and reared in a common pond, individual parental contributions and pedigree relationships were unavailable. The observed structure may partly reflect unequal parental contributions or familial relatedness, but the four exploratory clusters cannot be assigned to specific families, source populations, or hybrid ancestry on the basis of these data. Therefore, PC1–PC5 and a genomic relatedness matrix were retained in the primary mixed models to account for population structure and relatedness.

### 3.2. Genome-Wide Association Analyses Identify Major Regions on LG13, LG14, and LG04

**Figure 2:**
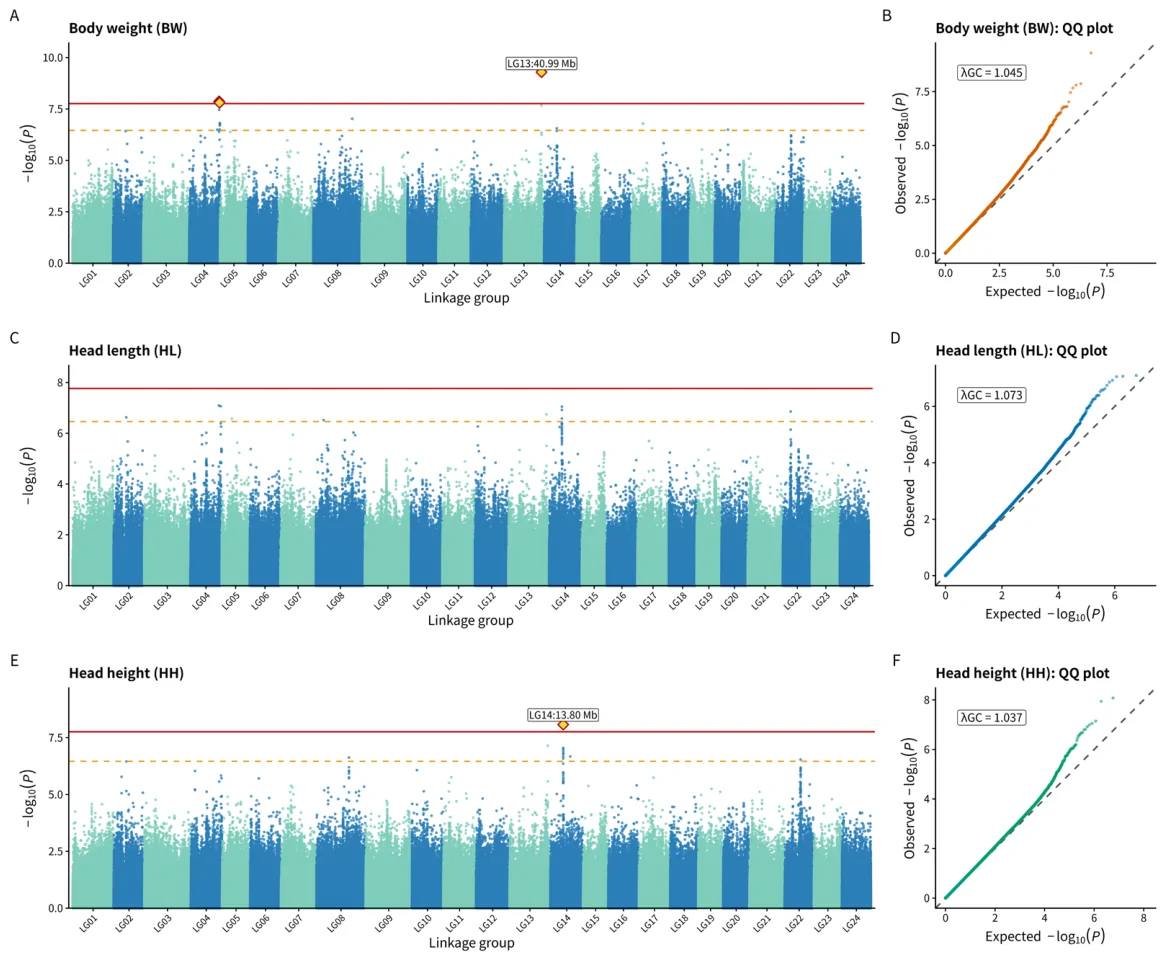
Genome-wide association analyses for body weight, head length, and head height in bighead carp. Manhattan and quantile–quantile (QQ) plots based on likelihood-ratio-test *p* values are shown for body weight (BW; **A**,**B**), head length (HL; **C**,**D**), and head height (HH; **E**,**F**). Association analyses were performed using a linear mixed model incorporating the first five principal components and a genomic relatedness matrix. Alternating blue and turquoise points in the Manhattan plots distinguish adjacent linkage groups. The solid red and dashed orange horizontal lines indicate the Bonferroni-corrected genome-wide significance threshold (*p* = 1.73 × 10^−8^) and the suggestive threshold (*p* = 3.46 × 10^−7^), respectively. Yellow diamonds with dark-red outlines indicate major lead SNPs. Genomic inflation factors (λGC) are shown in the QQ plots.

Before biallelic-SNP restriction, joint genotyping produced 22,556,132 variant records, including 12,142,734 SNPs. The analysis-ready PLINK dataset contained 3,863,038 SNPs in 298 fish, of which 2,891,492 entered each primary GEMMA analysis. All tested SNPs returned valid positive *p* values for BW, TL, BL, HL, and HH. Their λGC values were 1.045, 1.033, 1.029, 1.073, and 1.037, respectively (Figure 2; Table S2).

Three SNPs reached genome-wide significance for BW (Figure 2; Table S3). The strongest association was at LG13:40,990,738 bp: each additional C allele was associated with 5.50 ± 0.87 g greater BW (β ± SE; *p*_LRT_ = 5.09 × 10^−10^). The same SNP showed suggestive associations with TL (*p*_LRT_ = 2.06 × 10^−8^), BL (3.63 × 10^−8^), HL (1.80 × 10^−7^), and HH (7.13 × 10^−8^). Neither TL nor BL had a genome-wide significant SNP.

The strongest HH association occurred on LG14 at 13,799,852 bp. The C allele had an estimated effect of 2.59 ± 0.44 mm (*p*_LRT_ = 8.34 × 10^−9^); the adjacent SNP at 13,799,854 bp was also significant (*p*_LRT_ = 1.14 × 10^−8^). The LG14 lead showed suggestive associations with BW (*p*_LRT_ = 2.76 × 10^−7^) and HL (8.90 × 10^−8^), indicating overlap with other absolute size measurements.

LG04 showed a broader BW association pattern. SNPs 4:32624853:G:T and 4:33112675:C:T reached genome-wide significance (*p*_LRT_ = 1.35 × 10^−8^ and 1.57 × 10^−8^), with effects of 5.30 ± 0.92 g per G allele and 3.97 ± 0.69 g per C allele, respectively. A third representative SNP, 4:30566806:A:T, was suggestively associated with HL (*β* = 3.40 ± 0.63 mm; *p*_LRT_ = 8.07 × 10^−8^) and BW (*p*_LRT_ = 3.23 × 10^−7^). No SNP reached genome-wide significance for raw HL. Overall, five SNPs reached genome-wide significance: three for BW and two for HH, distributed across three chromosomal regions on LG13, LG04, and LG14. These five significant SNPs should not be interpreted as five independent association signals. In particular, the two significant HH SNPs on LG14 were separated by only 2 bp and exhibited complete LD (*r*^2^ = 1 among jointly genotyped individuals), consistent with a single regional association signal. The number of independent association signals within the more complex LG04 region remains unresolved.

The estimated effects of the same coded alleles were positive for both BW and logBW at all five representative lead SNPs, although association evidence was weaker after natural-log transformation. The LG13 lead remained suggestively associated with logBW (*β* = 0.395 ± 0.073, *p*_LRT_ = 1.04 × 10^−7^), as did LG04-B (*β* = 0.399 ± 0.077, *p*_LRT_ = 3.20 × 10^−7^). The corresponding *p*_LRT_ values were 4.23 × 10^−7^ for LG14, 7.25 × 10^−7^ for LG04-A, and 4.01 × 10^−7^ for LG04-C, which did not meet the suggestive threshold. None of these five SNPs reached genome-wide significance for logBW (Figure S2).

The final standardized HBR analysis yielded no genome-wide significant SNPs. Four SNPs reached the suggestive threshold, with the strongest association at LG18:15,591,042 bp (*p*_LRT_ = 4.49 × 10^−8^). A total of 2,891,490 SNPs returned finite *p* values, and λGC was 1.149. Given the residual genomic inflation, these suggestive associations were considered exploratory (Tables S2 and S3).

### 3.3. Regional LD and Conditional Analyses Refine the Association Architecture

**Figure 3:**
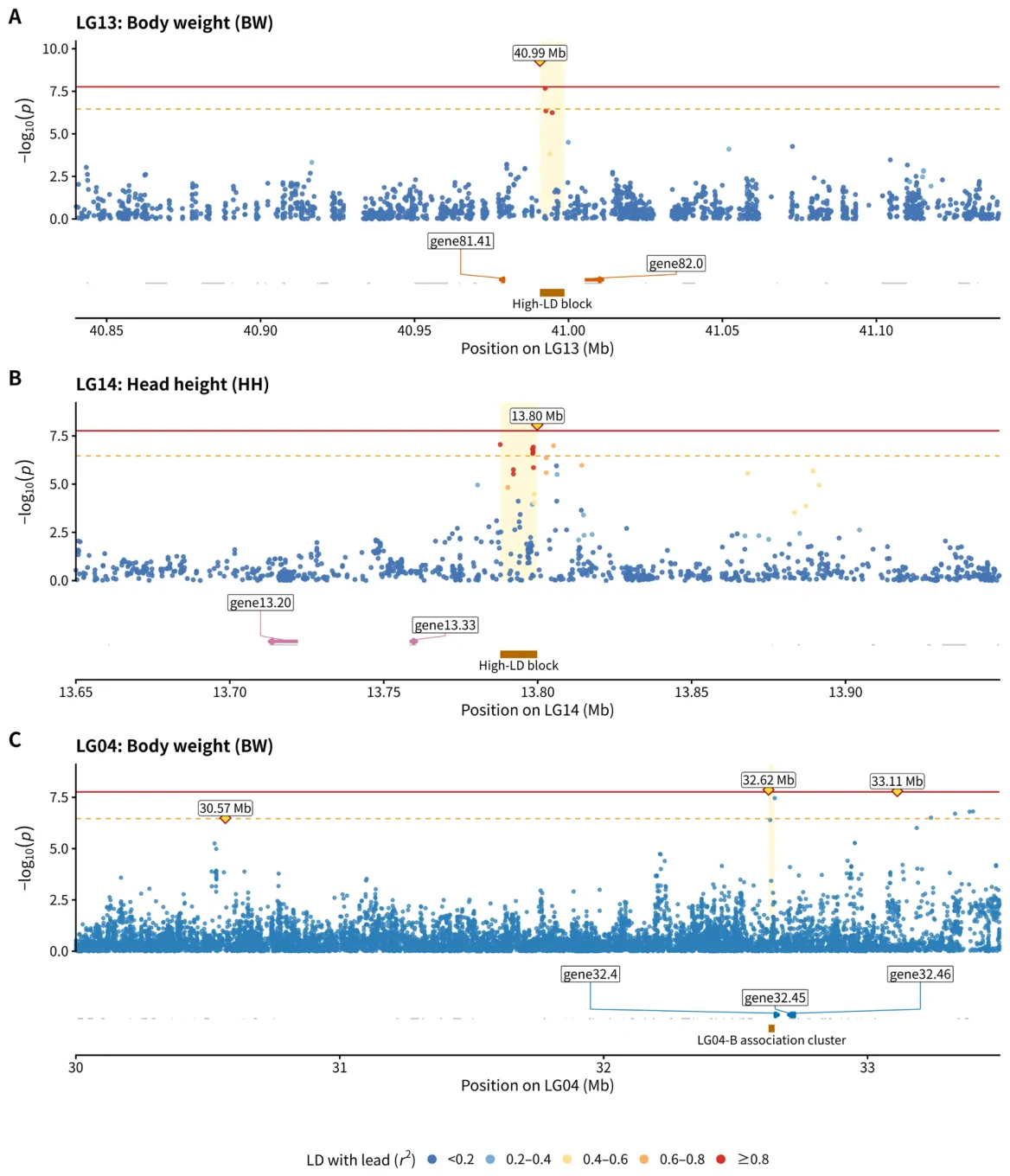
Regional association patterns and genomic context of major growth-associated loci in bighead carp. (**A**) Regional association plot for body weight around the lead locus on LG13. (**B**) Regional association plot for head height around the lead locus on LG14. In panels (**A**,**B**), point colors indicate linkage disequilibrium (LD; *r*^2^) with the corresponding lead SNP: blue represents low LD, yellow and orange represent intermediate LD, and red represents high LD. Pale-yellow vertical bands denote the lead-centered high-LD intervals. (**C**) Broad regional association profile of the complex LG04 locus for body weight. The pale-yellow vertical band marks the approximately 23 kb LG04-B local association cluster. Solid red and dashed orange horizontal lines indicate the genome-wide significance and suggestive thresholds, respectively. Yellow diamonds with dark-red outlines indicate major lead SNPs. Grey segments below the association plots represent surrounding gene models, whereas colored arrows indicate selected candidate genes and their transcriptional orientations.

The LG13 lead BW SNP lay within a compact high-LD interval extending from 40,990,738 to 40,998,720 bp, a span of 7983 bp (Figure 3; Table S5). Five additional variants had *r*^2^ ≥ 0.8 with the lead, and the highest non-self *r*^2^ was 0.874. The lead was intergenic: gene81.41 was 11.7 kb from the lead SNP and gene82.0 was 14.5 kb away. Conditioning BW on the lead SNP at 40,990,738 bp markedly attenuated the regional association. Within the LG13 window of 40,840,000–41,130,000 bp, the minimum *p*_LRT_ increased from 5.09 × 10^−10^ to 1.08 × 10^−3^, with the strongest remaining association at 40,966,586 bp (Figure S5; Table S4). No remaining SNP reached the per-trait suggestive or genome-wide significance threshold. These results are consistent with one predominant detectable association signal in this window, without establishing the number of causal variants.

Sequence-based annotation of the LG13 lead-centered high-LD interval showed that the 7983 bp region (LG13:40,990,738–40,998,720; GC content, 39.55%) contained no overlapping features in the reference GFF3 annotation. DUST identified 342 bp (4.28%) of low-complexity sequence, while TRF detected two tandem-repeat regions covering 327 bp (4.10%); their combined nonredundant coverage was 577 bp (7.23%). RepeatMasker, using curated and uncurated Dfam families, annotated 7245 bp (90.76%) as interspersed repeats, including LINEs (19.22%), LTR elements (28.08%), DNA transposons (28.11%), and unclassified repeats (15.35%). Overall, 7396 bp (92.65%) were masked when simple repeats and low-complexity sequences were also considered. Among the six SNPs with *r*^2^ ≥ 0.8 relative to the LG13 lead, four overlapped predicted repeat elements: positions 40,992,418 and 40,992,651 were located within the same unclassified repeat, position 40,994,230 overlapped an LTR annotation, and position 40,994,726 overlapped a DNA/CMC-EnSpm annotation. Neither the lead SNP at 40,990,738 bp nor the SNP at 40,998,720 bp overlapped a RepeatMasker annotation, and none of the six SNPs fell within DUST- or TRF-detected intervals. The high interspersed-repeat coverage depended on inclusion of uncurated Dfam families and should therefore be interpreted as computational annotation rather than definitive identification of functional transposable elements.

The LG14 lead-centered high-LD interval extended from 13,787,821 to 13,799,854 bp (12,034 bp, inclusive), with ten additional SNPs at *r*^2^ ≥ 0.8 to the lead. The two significant HH SNPs had *r*^2^ = 1 among 297 jointly genotyped fish. With one missing call at the lead, mean-imputed dosages remained nearly collinear (*r*^2^ = 0.99927); the adjacent SNP’s conditional coefficient was therefore not interpreted independently. Conditioning on the LG14 lead increased the minimum regional *p*_LRT_ from 8.34 × 10^−9^ to 1.20 × 10^−3^, with the strongest residual association at 13,651,375 bp and no residual suggestive or genome-wide significant SNP (Figure S5; Table S4). In contrast, conditioning on the LG13 lead retained the LG14 HH association (*β* = 2.50 ± 0.42 mm; *p*_LRT_ = 5.29 × 10^−9^).

LG04 contained three representative peaks at 30.57, 32.62, and 33.11 Mb with low pairwise LD (*r*^2^ = 0.133, 0.067, and 0.033). None had another marker at *r*^2^ ≥ 0.8 in its regional LD file. Around LG04-B, neighboring SNPs at 32,631,089 and 32,648,275 bp had *r*^2^ values of 0.655 and 0.510, respectively, illustrating a less compact LD pattern than at LG13 or LG14.

Mutual conditioning reduced the LG04 BW effects but retained positive estimates. When conditioned on the other two representatives, LG04-A, LG04-B, and LG04-C had effects of 2.44 ± 0.82 g (*p*_LRT_ = 2.57 × 10^−3^), 3.87 ± 0.91 g (2.12 × 10^−5^), and 3.02 ± 0.68 g (8.34 × 10^−6^), respectively. These nominal associations did not reach the adopted suggestive threshold. Joint conditioning on all three increased the regional minimum *p*_LRT_ from 1.35 × 10^−8^ to 8.85 × 10^−4^, at 32,754,867 bp (Figure S5; Table S4). No residual SNP met the suggestive or genome-wide threshold. The results identify a complex regional pattern without resolving the number of causal effects.

The number of neighboring SNPs meeting association thresholds varied considerably among the regions. Within ±50 kb of the LG13 BW lead, 932 neighboring SNPs had valid association results, of which three had *p*_LRT_ < 1 × 10^−5^ and one reached the suggestive threshold (*p*_LRT_ = 2.13 × 10^−8^), but none reached genome-wide significance. Around the LG14 HH lead, 395 neighboring SNPs were tested; 16 had *p*_LRT_ < 1 × 10^−5^, including eight at the suggestive threshold and one additional genome-wide significant SNP located 2 bp from the lead. For the LG04 BW signals, the numbers of neighboring SNPs with *p*_LRT_ < 1 × 10^−5^ were one, two, and zero within ±50 kb of LG04-A, LG04-B, and LG04-C, respectively. Only LG04-B had an additional suggestive SNP (*p*_LRT_ = 3.46 × 10^−8^), whereas neither LG04-A nor LG04-C had a suggestive neighboring SNP. These counts distinguish local association support from LD-based tagging and indicate that statistical support is uneven across the identified regions.

Bayesian fine-mapping showed variable resolution among the three associated regions. All three SuSiE models converged. At LG13, one purity-qualified 95% credible set contained two SNPs, with a minimum absolute within-set correlation of 0.855 and an estimated posterior coverage of 0.992. The lead SNP, 13:40990738:C:T, had a PIP of 0.941, whereas the second SNP, 13:40992418:T:G, had a PIP of 0.0528. Two additional highly diffuse unfiltered sets, each containing 1843 SNPs, failed the purity criterion and were not interpreted as independently localized signals. At LG14, one purity-qualified 95% credible set contained ten SNPs (minimum absolute correlation = 0.780). The two adjacent significant HH SNPs, 14:13799852:C:A and 14:13799854:T:C, had PIPs of 0.381 and 0.295, respectively, indicating limited resolution between these highly correlated variants. At LG04, SuSiE generated a nominal 95% credible set containing seven SNPs, with a sum of member PIPs of approximately 0.960. However, its minimum absolute within-set correlation was only 0.155, below the prespecified purity threshold of 0.5; consequently, no LG04 credible set passed purity filtering. The highest PIPs were observed for 4:32624853:G:T (0.410), 4:33112675:C:T (0.231), and 4:32648275:T:C (0.224), whereas 4:30566806:A:T had a PIP of 0.0349. Because the seven-SNP set did not meet the purity requirement, it was treated as an unfiltered, low-purity result rather than a well-localized credible set. These results did not establish the number or identity of independent causal variants within LG04.

Rare-homozygote exclusion analyses revealed different levels of robustness among the three LG04 representative SNPs. In the original 298-individual models conditioning on the other two LG04 SNPs, the estimated BW effects were +2.44 ± 0.82 g for LG04-A, +3.87 ± 0.91 g for LG04-B, and +3.02 ± 0.68 g for LG04-C. After excluding the five unique individuals carrying the rare AA genotype at LG04-A or the rare CC genotype at LG04-C, the corresponding estimates were +2.74 ± 0.94 g (*p*_LRT_ = 0.00323), +3.82 ± 0.93 g (*p*_LRT_ = 3.90 × 10^−5^), and +2.03 ± 0.75 g (*p*_LRT_ = 0.00626), respectively. These represented changes of +12.0%, −1.5%, and −32.8% relative to the original estimates. Excluding only the three rare CC homozygotes at LG04-C reduced its conditional effect from +3.02 to +2.16 g (*p*_LRT_ = 0.00340). Thus, LG04-A and LG04-B were comparatively stable under these exclusions, whereas LG04-C was more sensitive to the rare homozygous genotype class. All three conditional associations retained positive effects and nominal significance (*p*_LRT_ < 0.05) after the combined exclusion, but none met the prespecified per-trait suggestive threshold (*p*_LRT_ ≤ 3.458 × 10^−7^).

### 3.4. Genotype–Phenotype Patterns and Sensitivity of the LG13 Association

**Figure 4:**
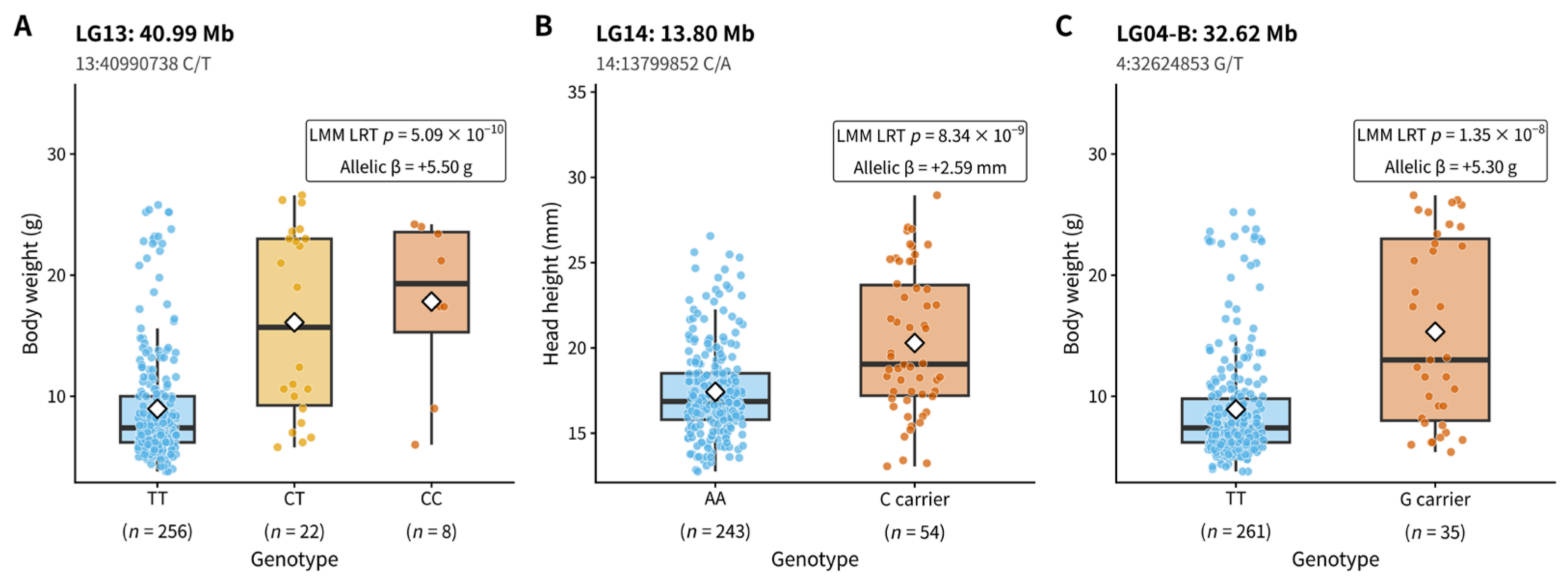
Phenotypic distributions according to lead-variant genotypes at major growth-associated loci in bighead carp. (**A**) Body weight according to genotype at the LG13 lead SNP 13:40990738 C/T. (**B**) Head height according to C-allele carrier status at the LG14 lead SNP 14:13799852 C/A. Carrier grouping was used because only one individual was homozygous for the C allele. (**C**) Body weight according to G-allele carrier status at the LG04-B lead SNP 4:32624853 G/T; no individuals homozygous for the G allele were observed. Blue denotes reference homozygotes, yellow denotes heterozygotes in panel (**A**), and orange denotes alternative homozygotes or carrier groups. Points represent individual fish; boxes show the interquartile range and median, whiskers extend to values within 1.5 times the interquartile range, and open diamonds indicate group means. Sample sizes are provided below the genotype labels, and individuals with missing genotypes were excluded from the corresponding panel. Allelic effect estimates (β) represent the per-copy effects of the counted C, C, and G alleles in panels (**A**–**C**), respectively. Effect estimates and likelihood-ratio-test *p* values were obtained from the GEMMA linear mixed model incorporating the first five principal components and the genomic relatedness matrix.

At the LG13 lead, mean BW was 8.97 g in TT fish (*n* = 256), 16.11 g in CT fish (*n* = 22), and 17.83 g in CC fish (*n* = 8), consistent with the positive additive effect (Figure 4; Table S6). All 30 C-allele carriers belonged to C3. Within this cluster, mean BW was 10.77 g in TT fish (*n* = 105), 16.11 g in CT fish, and 17.83 g in CC fish; three genotypes were missing.

Consistent with the cluster-level BW differences, fish in C3 had a higher mean BW than those in C1, C2, and C4 combined (11.97 ± 6.81 g, *n* = 138, versus 7.70 ± 2.70 g, *n* = 160; mean difference = 4.27 g; Welch’s *p* = 9.13 × 10^−11^). Importantly, this difference persisted when the comparison was restricted to TT homozygotes at the LG13 lead SNP. Mean BW among TT fish was 10.77 ± 6.11 g in C3 (*n* = 105), compared with 7.72 ± 2.75 g in the other three clusters combined (*n* = 151), corresponding to a mean difference of 3.05 g (95% CI: 1.79–4.31 g; Welch’s *p* = 4.33 × 10^−6^). Mean BW among TT individuals was 7.29 ± 2.41 g in C1 (*n* = 55), 7.72 ± 2.79 g in C2 (*n* = 67), and 8.53 ± 3.14 g in C4 (*n* = 29). Within C3, the 30 CT/CC carriers had a higher mean BW than the 105 TT individuals (16.57 ± 7.49 versus 10.77 ± 6.11 g; mean difference = 5.80 g; 95% CI: 2.78–8.81 g; Welch’s *p* = 3.70 × 10^−4^). These unadjusted comparisons indicate that both cluster-associated background variation and within-C3 genotype-associated differences contribute to the observed BW patterns, without resolving their causal contributions.

In full-cohort LOCO models, adding cluster indicators changed the BW effect little: 5.50 ± 0.87 g with PC adjustment alone versus 5.43 ± 0.87 g with PCs and clusters (*p*_LRT_ = 5.09 × 10^−10^ and 7.00 × 10^−10^). Within C3, effects remained positive for BW (5.73 ± 1.17 g; *p*_LRT_ = 1.58 × 10^−6^) and ln(BW) (0.416 ± 0.090; *p*_LRT_ = 5.36 × 10^−6^). These results provide within-cohort sensitivity evidence beyond comparisons among clusters.

Masking calls with DP < 4 or GQ < 10 increased missing genotypes from 12 to 23 and retained a BW effect of 5.38 ± 0.87 g (*p*_LRT_ = 1.14 × 10^−9^). The stricter DP < 5 or GQ < 20 rule left 198 TT, 20 CT, and six CC calls, with 74 missing genotypes (24.83%); the BW effect was 5.10 ± 0.98 g (*p*_LRT_ = 2.30 × 10^−7^). The corresponding ln(BW) effects were 0.386 ± 0.074 (*p*_LRT_ = 2.04 × 10^−7^) and 0.365 ± 0.083 (*p*_LRT_ = 1.04 × 10^−5^). Effect estimates were directionally consistent, while the stricter filter weakened statistical evidence.

Sanger sequencing provided an orthogonal check of the underlying LG13 calls. All 118 selected discovery-cohort fish were successfully typed as 88 TT, 22 CT, and eight CC, with complete agreement with the original whole-genome-sequencing calls (118/118; 100% concordance; exact 95% confidence interval, 96.9%–100%). This included all 62 calls removed only by the stringent DP/GQ masking rule. Thus, the loss of significance under that rule primarily reflected reduced genotype availability rather than observed discordance in the retyped samples.

At the LG14 HH lead, only one CC homozygote was observed. For visualization, AC and CC fish were therefore grouped as C-allele carriers. Mean HH was 17.43 mm in AA fish (*n* = 243) and 20.29 mm in C-allele carriers (*n* = 54), consistent with the GEMMA effect of +2.59 mm (*p*_LRT_ = 8.34 × 10^−9^). At LG04-B, no GG homozygotes were present; mean BW was 8.92 g in TT fish (*n* = 261) and 15.33 g in G-allele carriers (*n* = 35), consistent with the +5.30 g mixed-model effect (*p*_LRT_ = 1.35 × 10^−8^).

The additional LG04-A and LG04-C loci showed comparable raw phenotype shifts (Figure S6). At LG04-A, mean HL was 25.19 mm for TT (*n* = 259), 29.09 mm for AT (*n* = 32), and 32.67 mm for AA (*n* = 3). At LG04-C, mean BW was 8.77 g for TT (*n* = 241), 12.90 g for CT (*n* = 50), and 25.07 g for CC (*n* = 3). The very small minor-allele homozygote groups at both loci preclude inference about dose–response or dominance.

### 3.5. Secondary Analyses of Head Dimensions Relative to Body Length

Both head dimensions were strongly associated with BL on the log scale (Figure S4). The OLS slope was 0.953 ± 0.015 for ln(HL) versus ln(BL) (β ± SE; *R*^2^ = 0.929) and 1.022 ± 0.026 for ln(HH) versus ln(BL) (*R*^2^ = 0.836). In tests against an isometric slope of 1, the HL slope was slightly but significantly below 1 (95% CI: 0.923–0.983; *p* = 0.00206), whereas the HH slope did not differ significantly from 1 (95% CI: 0.971–1.074; *p* = 0.395). These cross-sectional relationships describe scaling among individuals at the sampled stage rather than individual growth trajectories.

In the secondary BL-adjusted scans, adjusted HL had 2,891,433 valid *p* values after numerical retries and 59 unavailable results; four SNPs were suggestive, led by LG18:15,591,042 (*p*_LRT_ = 5.53 × 10^−8^; λGC = 1.237). Adjusted HH had 2,891,492 valid results and one suggestive SNP at LG23:360,563 (*p*_LRT_ = 2.87 × 10^−7^; λGC = 0.968). Neither adjusted trait had a genome-wide significant SNP (Figure S3; Table S2). These exploratory scans address head dimensions conditional on BL.

Adding ln(BL) reduced estimated effects at all five representative loci when the log-transformed outcome, PCs, and relatedness matrix were otherwise held constant. At LG13, the ln(HL) effect decreased from 0.12437 ± 0.02497 to 0.00211 ± 0.00659, and the ln(HH) effect from 0.13980 ± 0.02840 to 0.00740 ± 0.01247; adjusted *p*_LRT_ values were 0.852 and 0.547. At LG14, the adjusted ln(HH) effect was 0.01946 ± 0.01034 (*p*_LRT_ = 0.0572). LG04-B retained nominal association with adjusted ln(HL) (*p*_LRT_ = 0.0227). None of these representative SNPs reached the suggestive threshold after adjustment (Figure S3; Table S2).

Additional adjustment for BW substantially attenuated the associations of the five representative SNPs with absolute HL and HH. At the LG13 lead, the estimated HL effect changed from +3.57 to −0.50 mm per C allele after BW adjustment (*p*_LRT_ = 0.0105). At the LG14 lead, the HH effect decreased from +2.59 to +0.58 mm per C allele (*p*_LRT_ = 0.00912). None of the five representative SNPs reached the per-trait suggestive threshold for either head dimension after BW adjustment. These results indicate substantial overlap between marker associations with absolute head dimensions and BW, without establishing whether the relationships are causal or mediated through body size.

### 3.6. Regional Annotation and Expression Context Prioritize Candidate Genes

**Figure 5:**
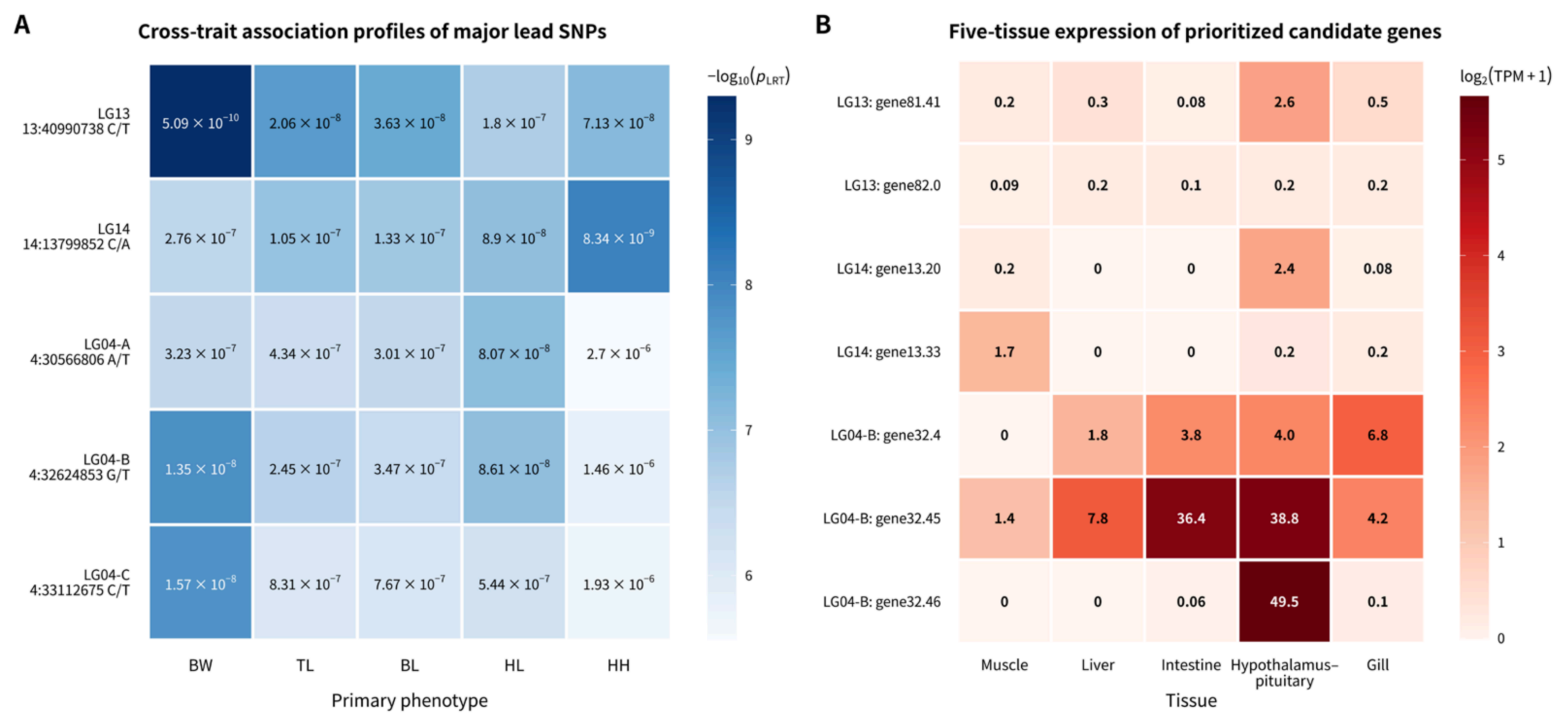
Cross-trait association profiles of major lead SNPs and descriptive tissue expression of prioritized candidate genes. (**A**) Likelihood-ratio test (LRT) association profiles for five representative lead SNPs across five primary growth-related phenotypes: body weight (BW), total length (TL), body length (BL), head length (HL), and head height (HH). Cell shading represents −log_10_(*p*_LRT_), and the numbers within the cells indicate the corresponding *p*_LRT_ values obtained from the GEMMA linear mixed models. (**B**) Descriptive expression profiles of seven prioritized candidate genes in muscle, liver, intestine, the hypothalamus–pituitary complex, and gill. Cell shading represents log_2_(mean TPM + 1), whereas the numbers within the cells indicate arithmetic mean TPM values calculated from three selected libraries per tissue group. Because these expression profiles integrate publicly available RNA-seq datasets generated from samples of different origins and experimental conditions, they should be interpreted as descriptive rather than as formal cross-tissue differential-expression comparisons.

Ten regional genes were summarized using genomic distance, protein similarity, and expression (Figure 3 and Figure 5; Tables S7 and S8). At LG13, gene81.41 and gene82.0 were 11.7 and 14.5 kb from the lead. The 403-aa gene82.0 model matched a *Phoxinus phoxinus* polymeric immunoglobulin receptor-like isoform (XP_079471822.1; 100% query coverage, 81.23% identity, BLAST-reported E value = 0.0), supporting a pIgR-like annotation. The gene81.41 remained unresolved. Both had low expression in the selected public libraries: gene81.41 reached 2.56 mean TPM in HP, whereas gene82.0 remained below 0.20 mean TPM in each group.

At LG14, gene13.33 and gene13.20 were 39.2 and 77.7 kb from the lead. The gene13.20 had a 307-aa predicted isoform and an MCHR2-like annotation supported by protein similarity. Its mean expression was highest in the selected HP group (2.40 TPM), with low or zero values in the other groups. The gene13.21 model had higher mean HP expression (144.53 TPM), but its short 72-aa model remained functionally unresolved.

At LG04-B, gene32.4, gene32.45, and gene32.46 were 36.5, 76.2, and 86.5 kb from the lead, respectively, and were annotated as C1QTNF1-like, SYNGR1-like, and CACNG2-like. The gene32.4 model showed its highest mean expression in the selected gill group (6.83 TPM); gene32.45 was expressed in intestine (36.44 TPM) and HP (38.81 TPM); and gene32.46 had 49.47 mean TPM in HP with low values elsewhere. At LG04-A, gene30.22 reached 545.42 mean TPM in HP, but its partial CDK5 activator 2a-like match remained tentative. These profiles describe the sampled tissue–condition groups rather than demonstrating tissue specificity.

Across the ten genes and three High-versus-Low comparisons, 11 candidate–comparison combinations had valid adjusted *p* values and 19 were excluded by the low-count prefilter. None of the 11 tested combinations met the differential-expression criteria (Table S8). For example, gene32.46 had an HP log2 fold change of −0.208 (*p* = 0.193; adjusted *p* = 0.999976), while gene32.45 was non-significant in HP, liver, and muscle. Prefiltered combinations were recorded separately as not tested. Expression evidence was therefore used to prioritize follow-up tissues and genes, not as confirmation of differential regulation by the associated variants.

### 3.7. Independent LG13 Association Replication and Genotype-Stratified Expression Analyses

**Figure 6:**
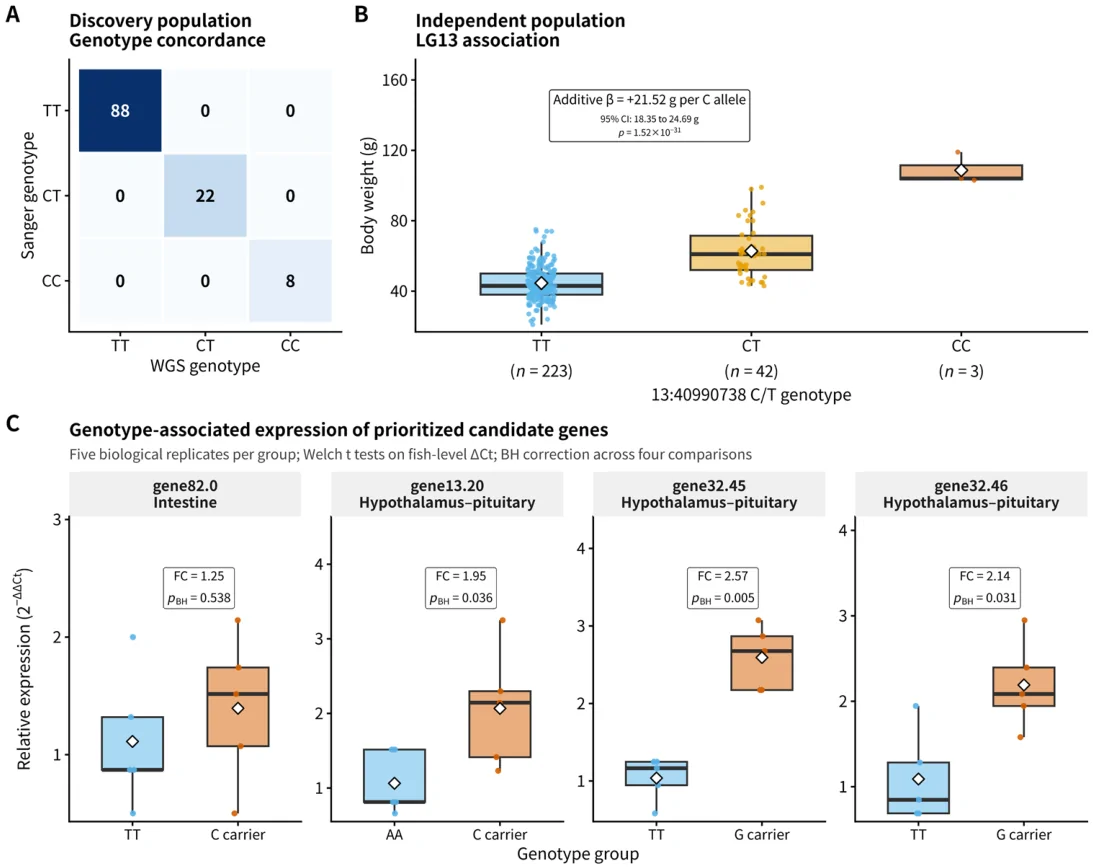
Orthogonal genotyping, independent LG13 association, and candidate-gene expression analyses in bighead carp. (**A**) Concordance between whole-genome sequencing (WGS) and Sanger genotypes at the LG13 lead SNP (13:40990738:C:T) in 118 discovery-cohort fish, including 62 samples with low sequencing depth or genotype quality. All tested genotypes were concordant. (**B**) Body weight according to LG13 genotype in the independent cohort (TT, *n* = 223; CT, *n* = 42; CC, *n* = 3). The displayed effect estimate and nominal *p* value were obtained from a single-marker additive ordinary least-squares regression model. This *p* value was not included in the RT-qPCR multiple-testing correction. (**C**) Genotype-stratified RT-qPCR analyses of four prioritized candidate genes in the indicated tissues. Each comparison included five biological replicates per genotype group, with three technical replicates averaged within each fish before statistical testing. The gene32.45 and gene32.46 assays used the same ten fish. In panel (**A**), darker blue indicates larger genotype counts. In panel (**B**), blue, yellow, and orange denote TT, CT, and CC genotypes, respectively. In panel (**C**), blue denotes reference-genotype fish (TT or AA), and orange denotes C- or G-allele carriers. Points represent individual fish, boxes show the median and interquartile range, and diamonds indicate group means. Two-sided Welch’s *t*-tests were performed using fish-level ΔCt values, and *p* values were adjusted using the Benjamini–Hochberg procedure across the four prespecified candidate-gene comparisons. The BH-adjusted *p* values are displayed in panel (**C**); corresponding unadjusted *p* values are reported in Section 3.7 and Table S9.

The genotyping concordance, independent-cohort association, and genotype-stratified expression results are summarized in Figure 6. The independent cohort contained 223 TT, 42 CT, and three CC fish at 13:40990738:C:T, corresponding to a C-allele frequency of 8.96%; genotype frequencies did not depart from Hardy–Weinberg expectations (exact *p* = 0.453). Mean BW was 44.58 ± 9.45 g in TT, 62.76 ± 15.24 g in CT, and 108.67 ± 8.96 g in CC fish. Because only three individuals carried the CC genotype, the corresponding genotype-specific mean BW and the apparent allele-dose pattern should be interpreted cautiously and should not be taken as reliable evidence of dominance or a precisely estimated homozygous effect. In the additive model, each C allele was associated with 21.52 ± 1.61 g greater BW (95% confidence interval, 18.35–24.69 g; *p* = 1.52 × 10^−31^; *R*^2^ = 0.402). The ln(BW) effect was 0.366 ± 0.032 per C allele (*p* = 6.62 × 10^−25^), equivalent to an estimated 44.1% multiplicative increase.

To contextualize the relatively large effect and R^2^ observed in the independent cohort, we compared the two cohorts using the same unadjusted single-marker OLS model. Among the 286 discovery fish with nonmissing LG13 genotypes, the C-allele effect on BW was +5.48 ± 0.72 g, with a sample R^2^ of 0.168, compared with +21.52 ± 1.61 g and R^2^ = 0.402 in the 268 independent fish. Mean BW differed substantially between cohorts (9.77 versus 48.15 g). On the log-transformed scale, the estimated effects were more similar: 0.424 ± 0.061 in the discovery cohort and 0.366 ± 0.032 in the independent cohort, corresponding to estimated multiplicative differences of 52.9% and 44.1% per C allele, respectively. In discovery-cohort OLS models, the partial R^2^ attributable to the SNP was 0.122 after PC1–PC5 adjustment and 0.119 after additional cluster adjustment; these conditional measures are not directly comparable with the unadjusted independent-cohort R^2^. Excluding the three CC homozygotes from the independent cohort reduced the BW effect to +18.18 ± 1.78 g and the sample R^2^ to 0.285 (*n* = 265). Thus, the small CC group contributed substantially to the unusually high independent-cohort R^2^, although a positive association persisted among TT and CT individuals. These R^2^ estimates describe cohort-specific model fit and should not be interpreted as proportions of genetic variance explained by the marker.

The same allele was positively associated with BL (+16.43 ± 1.48 mm; *p* = 9.35 × 10^−24^), HL (+5.40 ± 0.59 mm; *p* = 1.08 × 10^−17^), and HH (+4.53 ± 0.39 mm; *p* = 3.99 × 10^−25^). The BW association persisted in a secondary model including BL (+4.76 ± 0.67 g; *p* = 1.25 × 10^−11^) and in the corresponding log-scale model including ln(BL) (effect = 0.0426 ± 0.0110; *p* = 1.32 × 10^−4^). With HC3 robust standard errors, the additive BW association remained significant (*p* = 2.64 × 10^−15^). Excluding the three CC fish also retained the association: CT fish averaged 18.18 g more than TT fish (Welch *p* = 1.59 × 10^−9^), and the BL-adjusted CT effect remained positive (+3.66 g; HC3 *p* = 0.0026). These targeted results reproduce the direction of the discovery effect in fish from different parents, year class, and pond, although the magnitude is specific to this validation cohort.

Importantly, only the LG13 lead SNP was genotyped in this independent cohort; thus, genome-wide population structure and genomic relatedness could not be incorporated into the replication analysis. The independent-cohort results provide additional support for the LG13 marker–BW association but do not constitute independent marker-level validation of the LG14 or LG04 associations.

In the genotype-stratified RT-qPCR assays, gene13.20 expression in HP was 1.95-fold higher in LG14 C-allele carriers than in AA fish (95% confidence interval, 1.10–3.44; *p* = 0.0272; adjusted *p* = 0.0362). At LG04-B, HP expression was 2.57-fold higher for gene32.45 (95% confidence interval, 1.73–3.82; *p* = 0.00121; adjusted *p* = 0.00482) and 2.14-fold higher for gene32.46 (95% confidence interval, 1.23–3.74; *p* = 0.0153; adjusted *p* = 0.0305) in G-allele carriers than in TT fish. Intestinal gene82.0 expression did not differ detectably between LG13 C-allele carriers and TT fish (1.25-fold; 95% confidence interval, 0.56–2.77; *p* = 0.538; adjusted *p* = 0.538). Across the RT-qPCR sample sets, *β-actin* Cq values ranged from 19.5 to 20.5. Mean differences between corresponding genotype groups were 0.04–0.24 cycles, with no statistically detectable differences in the four comparisons (Welch’s *t*-tests, *p* = 0.210–0.667). These results indicated relatively consistent reference-gene Cq values within the examined sample sets. Body-weight records were available for all 15 individually identified fish used in the genotype-stratified RT-qPCR study. For gene13.20, mean BW was 46.84 ± 13.44 g in AA fish (*n* = 5) and 47.52 ± 9.21 g in C-allele carriers (*n* = 5), with no detectable difference between groups (Welch’s *t*-test, *p* = 0.9282). For gene32.45 and gene32.46, which were assayed in the same ten fish, mean BW was 58.82 ± 11.63 g in TT fish and 44.54 ± 11.94 g in G-allele carriers (*n* = 5 per group; *p* = 0.0918). For gene82.0, mean BW was 44.54 ± 10.36 g in TT fish and 49.08 ± 14.24 g in C-allele carriers (*n* = 5 per group; *p* = 0.5815). Values are means ± standard deviations. These descriptive comparisons provide information on body-size differences between expression groups but have limited power because of the small sample sizes.

## 4. Discussion

### 4.1. Whole-Genome Mapping Extends the Evidence for Size-Associated Regions in Bighead Carp

The principal contribution of this study is the identification and regional characterization of BW- and HH-associated regions on LG13, LG04, and LG14, together with follow-up analyses providing different levels of evidence for these regions. At LG13, Sanger sequencing confirmed the lead-SNP genotypes in selected discovery-cohort fish, and the positive marker–BW association was reproduced in an independently sampled cohort. In contrast, the LG14 and LG04 associations have not been independently replicated at the marker level. Genotype-stratified RT-qPCR analyses identified expression differences involving the LG14 candidate gene13.20 and the LG04 candidate genes gene32.45 and gene32.46, but these findings represent candidate-gene expression associations rather than independent replication of the corresponding GWAS signals. Regional LD, conditional association analyses, and Bayesian fine-mapping further characterized the association patterns and their remaining uncertainty without establishing causal variants. Thus, the available evidence supports a distinction between technical genotype confirmation, independent marker replication, candidate-gene expression associations, and statistical characterization. The LG14 and LG04 loci should be regarded as discovery-stage associations requiring independent marker-level replication and functional investigation.

Earlier bighead carp studies progressed from linkage mapping and fine mapping [6,7] to population-based association analyses. Zhou et al. mapped head-size and head-shape traits in a larger 2b-RAD cohort [2], and Chen et al. detected growth and body-conformation associations concentrated on LG16 and LG21 [3]. Our whole-genome dataset samples local variation densely around the detected peaks, complementing those reduced-representation studies. However, greater marker density does not compensate for the smaller number of fish or establish higher mapping power. The LG13, LG14, and LG04 findings should be evaluated alongside the earlier loci rather than treated as replacements. Determining whether intervals are shared requires direct coordinate harmonization and marker comparison across reference assemblies.

The overlap among absolute size traits has parallels in other cyprinids. In 200 rice flower carp, Luo et al. reported strong correlations among seven growth-related measurements and markers associated with several traits, particularly in regions on A8 and B25 [31]. In common carp, Chen et al. combined GWAS and linkage mapping and identified candidate regions involving both head dimensions and body growth, including an *igf1*-related interval [9]. Our LG13 lead similarly associated with BW and several linear measurements. Across other cultured fishes, the reported architecture ranges from chromosome-specific growth signals in Atlantic salmon to largely polygenic juvenile weight and length in a high-density salmon dataset [32,33]. Nile tilapia studies have likewise combined locus discovery with genomic prediction and have used multi-trait models to identify shared body-trait signals [34,35]. These comparisons show that a major regional association and a polygenic background are not mutually exclusive. They also caution against ranking studies by the number of significant SNPs alone, because marker density, sample size, LD, phenotype definition, and population history jointly determine the observed pattern.

### 4.2. LG13 Provides a Focused Body-Weight Association Target

LG13 was the strongest BW-associated region in the discovery cohort, with a lead *p*_LRT_ of 5.09 × 10^−10^. However, all observed C-allele carriers belonged to C3, and the TT homozygotes within C3 were also significantly heavier than TT homozygotes in the other clusters. This indicates that genetic background associated with cluster membership contributes to BW variation and represents a potential source of confounding. Additional adjustment for cluster membership changed the estimated LG13 effect only slightly, and a positive association persisted in the C3-specific LOCO mixed model (β = 5.73 ± 1.17 g; *p*_LRT_ = 1.58 × 10^−6^). The within-C3 result provides evidence that the marker association is not explained solely by the difference in mean BW between C3 and the other clusters. Nevertheless, because the C allele was absent from the other clusters and individual pedigree relationships were unavailable, these analyses cannot fully exclude residual familial confounding or establish that the allele has the same effect across genetic backgrounds. Complete Sanger concordance in 118 selected fish, including all 62 calls removed by stringent DP/GQ masking, reduces concern that the signal was created by systematic genotype-call error. The same C allele showed a positive association with BW in an independently sampled cohort of 268 fish from a different breeding batch and pond. The association remained positive after excluding the three CC homozygotes and after adjustment for BL. However, pedigree information was unavailable, preventing explicit adjustment for within-cohort family relatedness. Consequently, the independent-cohort results provide supporting association evidence but cannot exclude confounding by family structure. Although the absolute BW effect was substantially larger in this cohort than in the discovery cohort, the corresponding ln(BW) effects were more similar. Differences in body size, age, parental background, rearing conditions, and statistical models may contribute to the observed effect-size differences. The absolute effect estimate should therefore not be regarded as generalizable across populations, and further validation in populations with documented pedigree or genome-wide genotype information is needed.

The substantially larger absolute BW effect in the independent cohort warrants cautious interpretation. Although the estimated effect was approximately fourfold greater in grams, mean BW in the independent cohort was also approximately fivefold higher than in the discovery cohort. The corresponding log-scale effect estimates were considerably closer, suggesting that differences in growth stage and phenotype scale may account for part of the absolute effect discrepancy. Nevertheless, the independent-cohort sample R^2^ of 0.402 was unusually large for a single growth-associated marker and was sensitive to the three CC homozygotes, decreasing to 0.285 after their exclusion. Neither estimate should be interpreted as the proportion of additive genetic variance explained or as evidence of generalizable predictive performance. Moreover, the lead SNP was selected from a genome-wide discovery scan, so its discovery-stage effect may be affected by winner’s curse. The observed differences between cohorts cannot distinguish selection-related inflation from population-specific linkage disequilibrium, genetic background, developmental stage, or environmental effects. Independent family-based studies with comparable phenotyping and genome-wide relatedness information are needed to evaluate effect stability and the marker’s practical predictive value.

The approximately 8 kb lead-centered high-LD interval and the marked attenuation after conditioning provide a tractable starting point for denser genotyping and haplotype analysis. This differs from the distributed growth architecture observed by Ali et al. in rainbow trout, where SNP-window and single-marker analyses identified associations across several chromosomes [36], and from the strongly polygenic juvenile growth pattern reported in Atlantic salmon [33]. Their phenotypes included weight gain or size measured under different production designs, whereas our discovery measurement describes size at one juvenile sampling stage. Importantly, the independent-cohort BW association remained significant after adjustment for BL, indicating that the replicated signal was not explained entirely by longer bodies in that cohort. This secondary model changes the biological question from absolute harvest size to mass conditional on length; it does not replace the absolute-BW analysis. Repeated measurements would establish whether LG13 predicts subsequent growth rate, condition, or final harvest weight as well as current BW.

The LG13 lead is located in an intergenic region between gene81.41 and gene82.0, approximately 11.7 and 14.5 kb from their nearest boundaries, respectively. Sequence-based annotation provided additional context for the approximately 8 kb high-LD interval. No annotated gene features overlapped this region, whereas RepeatMasker classified a substantial proportion of its sequence as repetitive, including putative retrotransposon- and DNA transposon-derived fragments. Four of the six high-LD SNPs overlapped predicted repeat elements, although the lead SNP itself did not. Importantly, the extensive repeat annotation was obtained only when uncurated Dfam families were included, highlighting uncertainty in the precise repeat classification. These findings identify a repeat-annotated intergenic region rather than a demonstrated regulatory element, and they do not establish that transposable elements contribute to the BW association. The lead variant had the highest posterior inclusion probability in the regional fine-mapping analysis, whereas another variant in the credible set overlapped an unclassified repetitive sequence; neither observation demonstrates a causal mechanism. Teleost pIgR participates in mucosal immune functions [37], which do not provide a direct mechanistic explanation for juvenile BW variation. Furthermore, the nearby candidate genes showed low expression in the selected public transcriptomes, and targeted RT-qPCR did not detect a significant intestinal expression difference for gene82.0 between LG13 genotype groups. The causal gene, if any, and the potential regulatory consequences of the associated variants therefore remain unresolved. Future work should prioritize validation of local haplotypes and candidate variants, assessment of locus-specific chromatin accessibility and transcriptional regulation, and functional assays in relevant tissues and developmental stages.

### 4.3. Head-Height Associations Depend on the Breeding Phenotype of Interest

The LG14 association is a positive result for absolute HH: two adjacent SNPs were genome-wide significant, the local peak was attenuated by conditioning on the lead, and the lead remained associated after conditioning on LG13. These findings identify LG14 as a region associated with absolute juvenile HH, but do not establish an effect on head dimensions independent of overall body size. The economic interpretation differs among fish species. Geng et al. studied head size in hybrid catfish in the context of processing yield and adjusted measurements for the cube root of body weight within families before GWAS [11]. Kong et al. instead used proportional body-shape traits in large yellow croaker to address consumer preference for slender fish [10]. In bighead carp, the value of the head makes absolute head dimensions relevant alongside overall body weight; a size-dependent association can therefore still concern a commercially relevant phenotype [2,3].

Our secondary ln(BL)-adjusted analyses provide important evidence that the observed head-dimension associations largely reflect variation shared with overall body size rather than demonstrated head-specific effects. At LG13, the associations with ln(HL) and ln(HH) were strongly attenuated after adjustment for ln(BL), with *p*_LRT_ values of 0.852 and 0.547, respectively. Similarly, the LG14 association with ln(HH) was substantially weakened after adjustment (β = 0.01946 ± 0.01034; *p*_LRT_ = 0.0572), and no longer reached even nominal significance. Furthermore, neither BL-adjusted head trait yielded a genome-wide significant association. These findings indicate that LG13 and LG14 cannot currently be considered head-specific loci and suggest that their unadjusted head-dimension associations are predominantly related to overall body size. Nevertheless, the absence of a significant conditional association does not establish that all genetic effects are mediated through BL, particularly because BL is a highly correlated and potentially genetically influenced trait and adjustment for heritable covariates can complicate causal interpretation [38]. The original associations remain relevant to absolute head dimensions, but they do not provide convincing evidence for independent effects on head proportions or shape. Accordingly, marker-assisted selection aimed at increasing head dimensions relative to body size would require independent validation using appropriately adjusted head traits, head-to-body ratios, or direct measurements of head yield.

Melanin-concentrating hormone signaling has been implicated in feeding regulation in fish [39,40]. However, the MCHR2-like protein annotation does not establish the biological function of gene13.20 in bighead carp. The *mchr2*-like gene model (gene13.20) remains an exploratory candidate for the LG14 region rather than an established effector gene. It is located approximately 77.7 kb from the lead SNP and outside the approximately 12 kb high-LD interval. Genotype-stratified RT-qPCR showed approximately 1.95-fold higher expression in C-allele carriers than in AA fish, but the expression comparison alone cannot establish a regulatory connection between the associated variant and gene13.20. Importantly, the two expression groups had very similar mean BW (46.84 ± 13.44 g for AA fish versus 47.52 ± 9.21 g for C-allele carriers; *n* = 5 per group; *p* = 0.9282), suggesting that the observed expression difference was not accompanied by an obvious difference in average body weight in these selected fish. However, the small sample size and wide individual variation limit the ability to exclude body-size-related effects. Furthermore, recent feeding, nutritional and physiological status, and differences in cellular composition within the mixed hypothalamus–pituitary tissue were not sufficiently controlled to establish a causal interpretation. Because the LG14 association with HH was substantially attenuated after adjustment for BL, the expression finding should not be taken as evidence of a head-specific neuroendocrine mechanism. The present data therefore support further investigation of gene13.20, but do not demonstrate that its expression mediates the LG14 association. Confirmation will require larger, phenotypically characterized groups, standardized feeding conditions, tissue-resolved expression analyses, and functional validation.

### 4.4. LG04 and Public Transcriptomes Define Additional Follow-Up Priorities

LG04 differs from the more compact LG13 and LG14 association patterns. Although its three representative SNPs exhibited low pairwise LD, their BW associations were attenuated by mutual conditioning, and joint conditioning removed regional associations meeting the adopted suggestive threshold. Bayesian fine-mapping generated a nominal seven-SNP 95% credible set, but this set had low purity (minimum absolute correlation = 0.155) and did not satisfy the prespecified threshold of 0.5. Consequently, the posterior evidence did not provide sufficient resolution to distinguish independent association signals or nominate a causal variant within LG04. The rare-homozygote exclusion analyses further demonstrated that the LG04-C conditional effect was sensitive to a small genotype class, decreasing by 32.8% after the combined exclusion of rare AA and CC individuals. In contrast, LG04-A and LG04-B showed comparatively smaller effect changes. The persistence of nominally significant conditional associations should therefore not be interpreted as confirmation of independent causal loci. Larger cohorts with more informative recombination events, better representation of minor genotype classes, and independent replication are needed to resolve this region. These limitations reflect the broader challenges of identifying and fine-mapping quantitative growth loci in aquaculture populations [4,8,14].

The regional candidates suggest metabolic and neuronal functions for experimental evaluation. The C1QTNF1-like gene32.4 belongs to a family implicated in metabolic regulation, although much of the mechanistic evidence comes from mammals [41]. Synaptogyrin family proteins participate in synaptic-vesicle biology and plasticity [42], while CACNG2/stargazin regulates AMPA-receptor trafficking and synaptic targeting [43]. These functions make gene32.45 and gene32.46 plausible components of neuroendocrine pathways influencing feeding or growth, but the cited mechanisms were established primarily in mammalian neural systems and cannot be transferred directly to bighead carp. In genotype-stratified RT-qPCR analyses of hypothalamus–pituitary (HP) samples, *syngr1*-like gene32.45 and *cacng2*-like gene32.46 showed approximately 2.57- and 2.14-fold higher expression, respectively, in LG04-B G-allele carriers than in TT fish, with both differences remaining statistically significant after Benjamini–Hochberg correction across the four prespecified comparisons. Among these candidates, gene32.45 showed the strongest statistical evidence for a genotype-associated expression difference. These findings strengthen their priority within the region, but the broad LG04 association pattern, low LD among representative SNPs, and small RT-qPCR groups preclude assigning causality to a particular variant or transcript. The body-weight comparison also warrants caution. The G-allele carriers used for the gene32.45 and gene32.46 expression assays had a lower mean BW than the TT fish (44.54 ± 11.94 versus 58.82 ± 11.63 g; *n* = 5 per group), although the difference did not reach nominal statistical significance (*p* = 0.0918). Given the small sample size and the magnitude of the observed difference, body-size-related or physiological influences on gene expression cannot be excluded. Moreover, the two genes were examined in the same ten fish, and their expression associations should not be interpreted as independent evidence of causal regulation.

Fu et al. used HP and liver transcriptomes from growth-extreme bighead carp and examined their overlap with growth-related QTL [15]. Our reanalysis of those public libraries found no significant differential expression among the evaluable regional candidates. This is not inconsistent with the new RT-qPCR results: the public contrasts were defined by growth group and came from other experiments, whereas the targeted assays compared genotype-stratified groups for prespecified genes. The genotype-associated increases for gene13.20, gene32.45, and gene32.46 therefore add focused evidence, while the public data retain value for tissue prioritization. Neither analysis alone establishes a regulatory path from the associated SNPs to phenotype.

### 4.5. Breeding Implications and Scope of Inference

The present results identify an externally replicated BW-associated marker on LG13 and discovery-stage candidate regions on LG14 and LG04. Although genotype-associated expression differences help prioritize genes in the latter regions, they do not independently validate the corresponding marker–trait associations. Therefore, LG14 and LG04 require replication of their marker effects in additional populations before their potential use in breeding can be assessed. Local association support was uneven, with relatively few neighboring SNPs meeting the suggestive threshold around LG13 and LG04-B and no such neighboring SNP around LG04-C within ±50 kb. These sparse regional signals, particularly those lacking external replication, warrant cautious interpretation and further validation in larger populations. A large-effect marker may be useful for low-cost screening, but it should be tested for allele frequency, effect stability, and possible correlated responses in each breeding population. Studies in Atlantic salmon and Nile tilapia show that genome-wide information can improve prediction for growth-related traits even when individual loci explain only part of the genetic variance [32,33,34,35]. Reviews of aquaculture genomic selection likewise emphasize population-specific training, adequate reference populations, and validation under the intended production conditions [4,5,44]. Thus, LG13 is better viewed as a candidate addition to genome-wide prediction or marker-assisted decision rules than as a stand-alone predictor. The present analyses do not estimate breeding values, selection response, or out-of-sample prediction accuracy.

The distinction between absolute head size and head dimensions relative to overall body size is particularly important for breeding applications. Although LG14 was associated with absolute HH, its markedly attenuated association after BL adjustment suggests that marker-based selection at this region may primarily track general body-size variation rather than selectively increase head height relative to body length. However, the present association analyses cannot predict the actual response to selection or establish that selecting the LG14 allele would necessarily increase BW. For breeding programs aiming to improve disproportionate head size or head yield, selection decisions should therefore consider BL-adjusted HH, head-to-body ratios, and direct head-yield measurements, together with absolute growth traits. Because LG14 has not undergone independent marker-level replication, its use in marker-assisted selection is premature. Validation across independent families and production stages, including assessment of correlated changes in BW, BL, and head proportions, is required before its breeding value can be established.

Several limits remain. The discovery cohort comprised 298 fish from one pond and one age, with unknown sex and pedigree, and minor-allele homozygote groups were small. Although the independent cohort used different parents and another pond, only the target LG13 marker was genotyped; genome-wide structure and relatedness could not be modeled, and only three CC fish were observed. The number of contributing families and individual parental assignments were not available at the level required for family-based association analysis; consequently, family-specific effects could not be estimated directly. The unusually large within-cohort R^2^ estimates, particularly in the independent cohort, should therefore be interpreted with caution because unobserved familial structure may contribute to the apparent marker effects. Sequential sampling avoided deliberate selection by phenotype, but it cannot rule out family structure or other unmeasured covariates. In particular, the complete restriction of the LG13 C allele to C3, together with the higher BW of TT homozygotes in that cluster, precludes complete separation of marker-associated effects from unrecorded familial or cluster-specific genetic background effects in the discovery cohort. Although *β-actin* Cq values were relatively consistent across the examined genotype groups, no alternative reference genes were evaluated. Therefore, this preliminary assessment does not establish reference-gene stability or exclude normalization-related bias. Each RT-qPCR comparison included only five biological replicates per genotype group, limiting statistical power and the precision of the estimated expression differences. Furthermore, some candidate-gene assays involved overlapping biological samples, with gene32.45 and gene32.46 examined in the same ten fish. Accordingly, these findings should be regarded as exploratory genotype-associated expression evidence rather than definitive functional validation. Future studies should validate multiple reference genes and confirm the expression patterns in larger, independently sampled populations. Effect estimates may also be inflated by discovery-stage selection [14]. The fine-mapping results were conditional on covariance parameters estimated from the original GEMMA null models and should be interpreted as model-dependent posterior summaries rather than definitive causal assignments. In particular, the low-purity seven-SNP credible set at LG04 and the sensitivity of LG04-C to rare homozygotes limit the resolution of independent genetic effects within this region. Replication of LG14 and LG04, multi-family tests of LG13, denser regional genotyping, validation of reference-gene stability, and perturbation or reporter assays would further resolve these limitations. Nevertheless, orthogonal genotyping, external LG13 replication, and targeted expression assays substantially strengthen the present evidence and define specific next experiments rather than leaving the GWAS signals untested.

## 5. Conclusions

Whole-genome association mapping identified BW-associated regions on LG13 and LG04 and a region associated with absolute, body-size-related HH on LG14 in juvenile bighead carp. The LG13 lead genotype was confirmed by Sanger sequencing in 118 discovery-cohort fish, and its positive BW association was independently replicated in a cohort of 268 fish from different parents and another pond, although genetic-background and family-related confounding cannot be fully excluded. In contrast, the LG14 and LG04 associations remain unreplicated at the marker level and should be considered preliminary findings requiring confirmation in independent populations. Importantly, the LG13 associations with head dimensions were no longer supported after adjustment for BL (*p*_LRT_ = 0.852 for HL and 0.547 for HH), while the LG14 HH association was markedly attenuated (*p*_LRT_ = 0.0572). These findings indicate that the observed head-dimension signals are largely associated with overall body size and do not establish head-specific genetic effects. Consequently, the LG14 marker should not be considered suitable for selection targeting head proportions without independent replication and direct evaluation of body-size-adjusted head traits. Regional LD analysis, conditional testing, and Bayesian fine-mapping provided additional information about association patterns and their resolution, but did not establish causal variants. Genotype-stratified RT-qPCR identified expression associations involving *mchr2*-like gene13.20, *syngr1*-like gene32.45, and *cacng2*-like gene32.46, whereas *pigr*-like gene82.0 showed no detectable intestinal expression difference. These expression results prioritize candidate genes for further investigation but do not constitute independent validation of the LG14 or LG04 association signals or demonstrate causal mechanisms. Overall, LG13 represents a replicated BW-associated marker warranting further evaluation, while LG14 and LG04 represent discovery-stage candidate regions requiring independent replication and functional studies before application in breeding.

## Data Availability

The raw whole-genome resequencing reads have been deposited in the NCBI Sequence Read Archive under BioProject PRJNA1524345. The public RNA-seq datasets reanalyzed in this study are available under BioProjects PRJNA1253371, PRJNA721309, PRJNA1303128, and PRJNA453809.

## Supplementary Materials

The following supporting information can be downloaded at https://www.mdpi.com/article/10.3390/biology15201896/s1. Figure S1: Scree plot of the first 20 principal components derived from 532,418 LD-pruned SNPs in 298 bighead carp; Figure S2: Genome-wide association analyses for total length, body length, and log-transformed body weight; Figure S3: Genome-wide association analyses of body-length-adjusted head traits; Figure S4: Allometric relationships and body-length adjustment of head dimensions in 298 bighead carp; Figure S5: Original and conditional regional association patterns at the major growth-associated loci; Figure S6: Phenotypic effects of two additional LG04 lead variants; Table S1: Phenotypic descriptive statistics, Pearson correlations, genetic differentiation among inferred subgroups, and subgroup body-weight comparisons; Table S2: Summary of GWAS analyses, SNP-based PVE estimates and standard errors, and pairwise genetic correlation estimates; Table S3: SNPs reaching the fixed genome-wide or suggestive thresholds in the final association analyses; Table S4: Original and conditional GWAS analyses and conditional sensitivity analyses at the major associated loci; Table S5: Linkage disequilibrium, neighboring SNP support, SuSiE fine-mapping results, and repeat-sequence annotations; Table S6: Genotype-group statistics, additive SNP effects, and LG13 population-structure and genotype-quality sensitivity analyses; Table S7: Genomic context, predicted protein lengths, and functional annotations of prioritized candidate genes; Table S8: Five-tissue mean TPM values and High-versus-Low DESeq2 results for candidate genes, including explicit low-expression prefilter status; Table S9: WGS–Sanger genotype concordance, independent-population validation, individual RT-qPCR data, and validation primer information. Supplementary Figures S1–S6 and their legends are provided in Supplementary_material.docx, and Supplementary Tables S1–S9 are supplied as nine separate Excel workbooks.

## Abbreviations

The following abbreviations are used in this manuscript:

AMPA	α-Amino-3-hydroxy-5-methyl-4-isoxazolepropionic acid
BL	Body length
BLASTP	Protein–protein Basic Local Alignment Search Tool
BW	Body weight
*c1qtnf1*	C1q and tumor necrosis factor-related protein 1
*cacng2*	Calcium voltage-gated channel auxiliary subunit gamma 2
cDNA	Complementary deoxyribonucleic acid
*cdk5*	Cyclin-dependent kinase 5
CI	Confidence interval
Ct	Cycle threshold
DNA	Deoxyribonucleic acid
DP	Read depth
*fndc5b*	Fibronectin type III domain-containing protein 5b
GATK	Genome Analysis Toolkit
GEMMA	Genome-wide Efficient Mixed Model Association
GQ	Genotype quality
gVCF	Genomic variant call format
GWAS	Genome-wide association study
HBR	Head-to-body length ratio
HC3	Heteroscedasticity-consistent covariance estimator, type 3
HH	Head height
HL	Head length
HP	Hypothalamus–pituitary complex
*igf1*	Insulin-like growth factor 1
IgM	Immunoglobulin M
LD	Linkage disequilibrium
LG	Linkage group
LMM	Linear mixed model
LOCO	Leave-one-chromosome-out
LRT	Likelihood-ratio test
MAF	Minor allele frequency
MCH	Melanin-concentrating hormone
*mchr2*	Melanin-concentrating hormone receptor 2
MS-222	Tricaine methanesulfonate
NCBI	National Center for Biotechnology Information
OD	Optical density
OLS	Ordinary least squares
PC	Principal component
PCA	Principal component analysis
PCR	Polymerase chain reaction
*pigr*	Polymeric immunoglobulin receptor
QQ	Quantile–quantile
QTL	Quantitative trait locus
RNA	Ribonucleic acid
RNA-seq	RNA sequencing
RT-qPCR	Reverse-transcription quantitative polymerase chain reaction
SD	Standard deviation
SE	Standard error
SNP	Single-nucleotide polymorphism
SRA	Sequence Read Archive
STAR	Spliced Transcripts Alignment to a Reference
*syngr1*	Synaptogyrin 1
TL	Total length
*tp53bp2*	Tumor protein p53 binding protein 2
TPM	Transcripts per million
VCF	Variant call format
WGS	Whole-genome sequencing
λGC	Genomic inflation factor
