# Supplementary material for: Whole-Genome Resequencing Identifies Loci Associated with Body Weight and Body-Size-Related Head Height in Bighead Carp (Hypophthalmichthys nobilis)
Source: Biology (Basel). 2026 Oct 21;15(20):1896. doi: 10.3390/biology15201896 (PMC13701794; doi:10.3390/biology15201896)
Supplement: Supplementary file 1 [file biology-15-01896-s001.zip › Supplementary_material.pdf]

Supplementary Materials

Whole-genome resequencing identifies loci associated with body weight and head height in bighead carp (*Hypophthalmichthys nobilis*)

This file contains Supplementary Figures S1-S6 and their complete legends. Supplementary Tables S1–S9 are supplied as separate machine-readable Excel files; their descriptions are provided after the figures.

Contents

1. Figure S1. Scree plot of the first 20 principal components derived from 532,418 LD-pruned SNPs in 298 bighead carp.
2. Figure S2. Genome-wide association analyses for total length, body length, and log-transformed body weight.
3. Figure S3. Genome-wide association analyses of body-length-adjusted head traits.
4. Figure S4. Allometric relationships and body-length adjustment of head dimensions in 298 bighead carp.
5. Figure S5. Original and conditional regional association patterns at the major growth-associated loci.
6. Figure S6. Phenotypic effects of two additional LG04 lead variants.
7. Tables S1-S9. Data tables and validation records supplied as separate files.

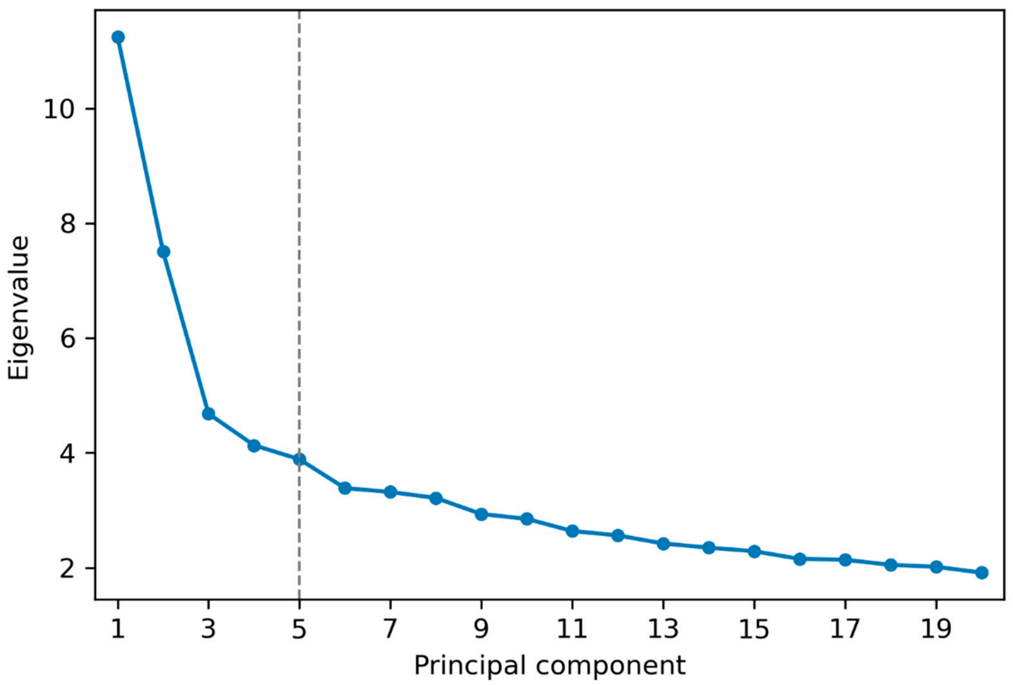

Figure S1. Scree plot of the first 20 principal components derived from 532,418 LD-pruned SNPs in 298 bighead carp. The dashed line indicates the first five PCs included as fixed-effect covariates in the GWAS models.

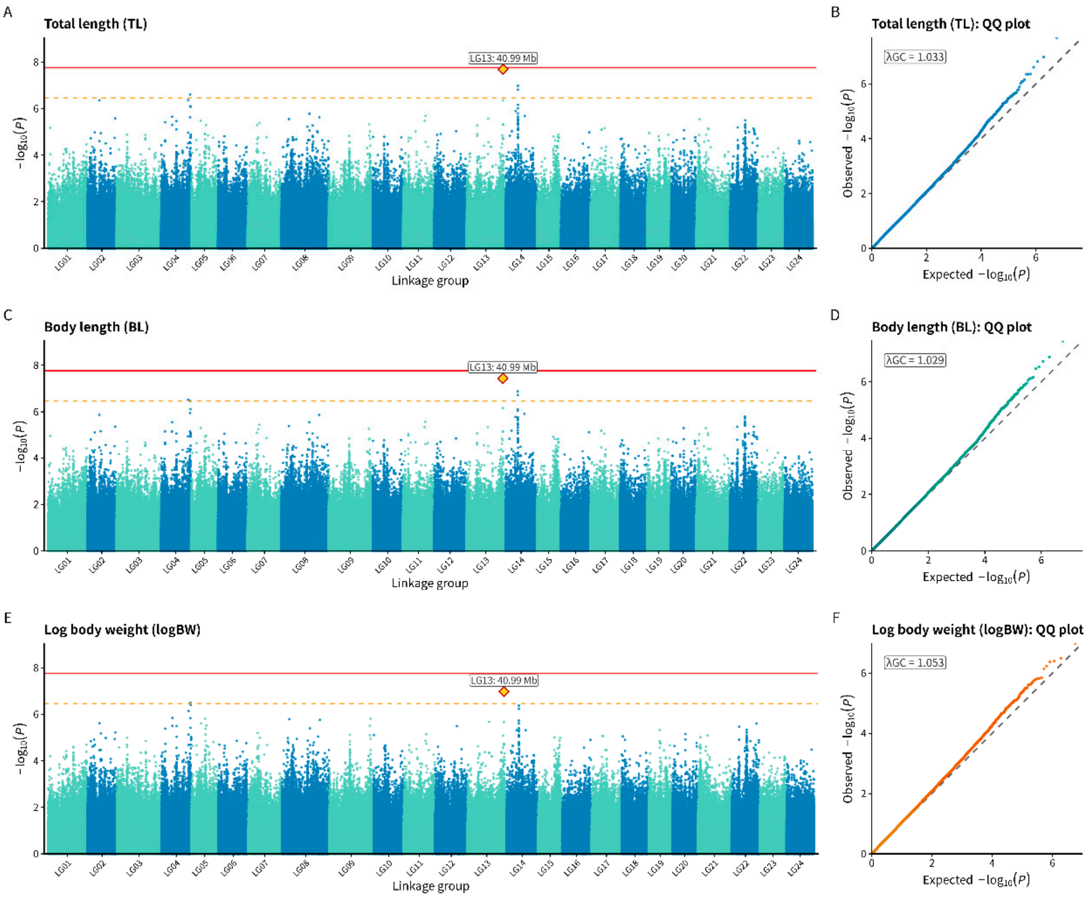

Figure S2. Genome-wide association analyses for total length, body length, and log-transformed body weight. Manhattan and quantile–quantile (QQ) plots are shown for total length (TL; A, B), body length (BL; C, D), and natural-log-transformed body weight (logBW; E, F). Analyses were performed using a linear mixed model incorporating the first five principal components and the genomic relatedness matrix. Solid red and dashed orange lines indicate the genome-wide significance and suggestive thresholds, respectively. Yellow diamonds mark the LG13 body-weight lead SNP (13:40990738 C/T) for cross-trait comparison. Genomic inflation factors (λGC) are shown in the QQ plots.

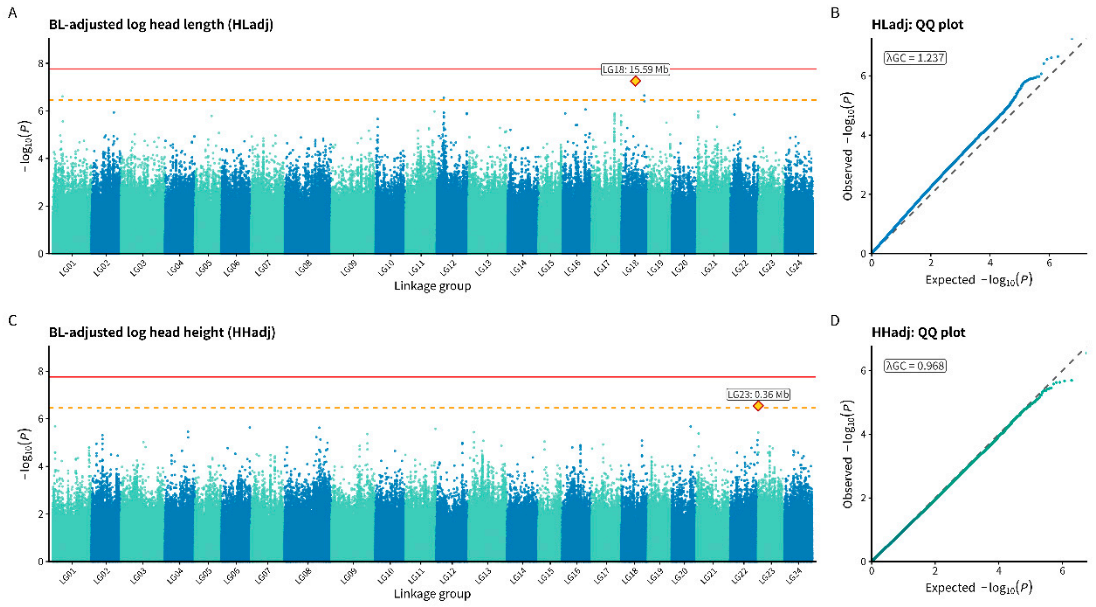

Figure S3. Genome-wide association analyses of body-length-adjusted head dimensions. Manhattan and quantile–quantile (QQ) plots are shown for log-transformed head length adjusted for log-transformed body length (HLadj; A, B) and log-transformed head height adjusted for log-transformed body length (HHadj; C, D). The linear mixed models additionally incorporated the first five principal components and the genomic relatedness matrix. Solid red and dashed orange lines indicate the genome-wide significance and suggestive thresholds, respectively. Diamonds mark the SNP with the lowest P value in each analysis, and genomic inflation factors (λGC) are shown in the QQ plots.

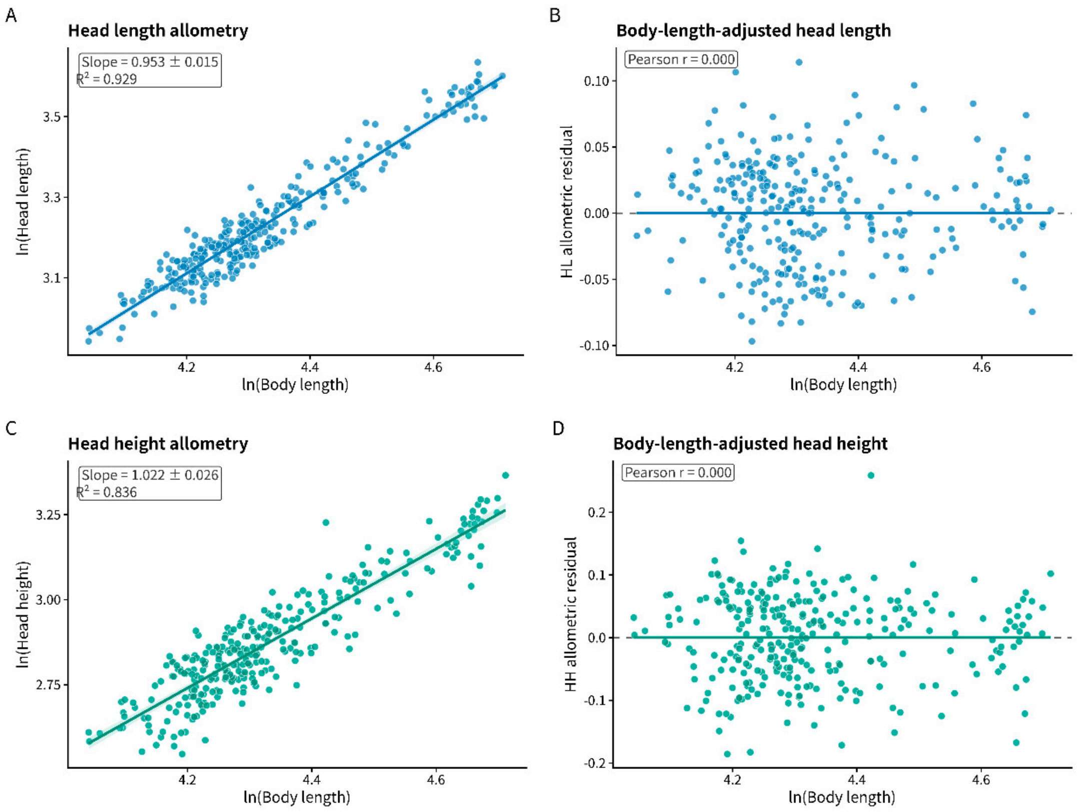

Figure S4. Allometric relationships of head dimensions with body length in bighead carp. Relationships of ln-transformed body length with head length (A) and head height (C), together with the corresponding allometric residuals (B, D), are shown for 298 fish. Solid lines and shaded bands in A and C represent linear regressions and 95% confidence intervals. Dashed horizontal lines in B and D indicate zero residuals. Insets report regression slopes ± standard errors, coefficients of determination (R^2), or Pearson correlation coefficients. Each point represents one individual.

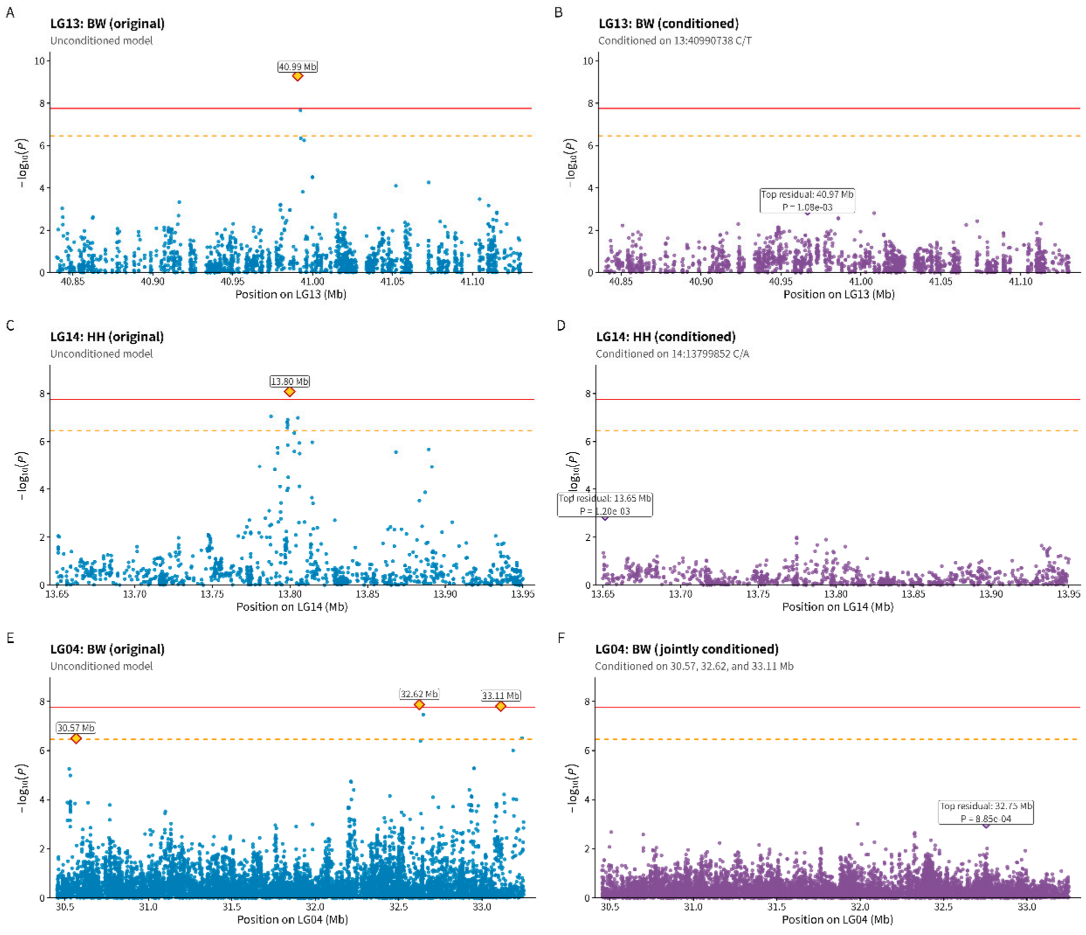

Figure S5. Conditional analyses of major growth-associated regions in bighead carp. Original and conditional regional association profiles are shown for body weight on LG13 (A, B), head height on LG14 (C, D), and body weight on LG04 (E, F). Models were conditioned on the LG13 lead SNP, the LG14 lead SNP, or jointly on three representative LG04 SNPs while retaining the first five principal components and the genomic relatedness matrix. Yellow diamonds mark the original lead SNPs, whereas purple diamonds indicate the strongest residual associations after conditioning. Solid red and dashed orange lines denote the genome-wide significance and suggestive thresholds, respectively.

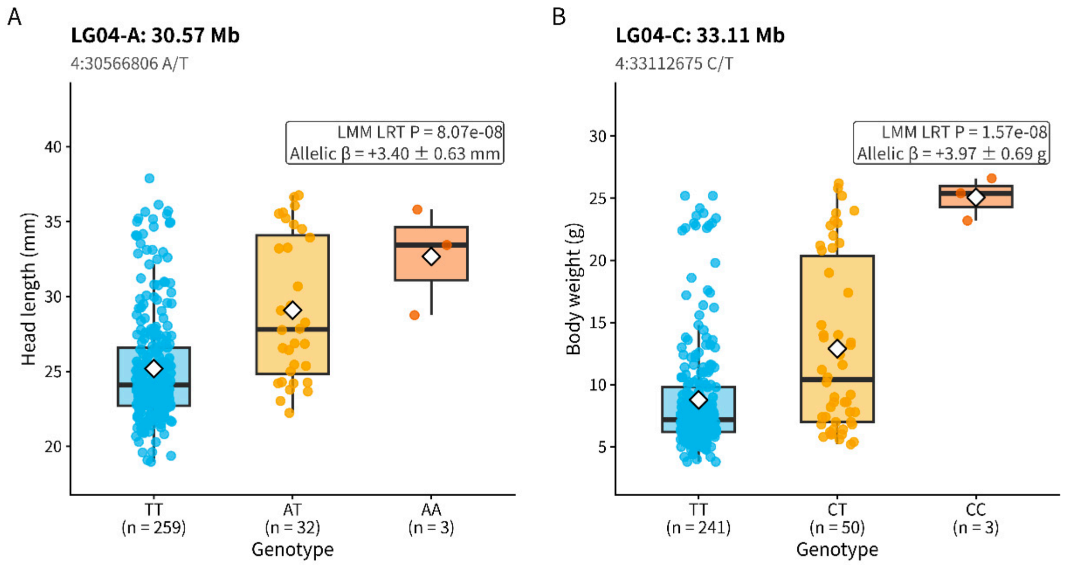

Figure S6. Phenotypic distributions across genotypes at two additional LG04-associated variants. Head length according to genotype at the LG04-A SNP 4:30566806 A/T (A) and body weight according to genotype at the LG04-C SNP 4:33112675 C/T (B). Points represent individual fish, boxes indicate the interquartile range with the median, and open diamonds indicate group means. Allelic effect estimates ± standard errors and likelihood-ratio-test P values were obtained from the GEMMA linear mixed models. The plotted genotype groups comprised 294 fish for each variant; group-specific sample sizes are indicated below the boxplots.

Supplementary Tables

**Table S1**	Phenotypic descriptive statistics, Pearson correlations, genetic differentiation among inferred subgroups, and subgroup body-weight comparisons.
**Table S2**	Summary of GWAS analyses, SNP-based PVE estimates and standard errors, and pairwise genetic correlation estimates.
**Table S3**	SNPs reaching the fixed genome-wide or suggestive thresholds in the final association analyses.
**Table S4**	Original and conditional GWAS analyses and conditional sensitivity analyses at the major associated loci.
**Table S5**	Linkage disequilibrium, neighboring SNP support, SuSiE fine-mapping results, and repeat-sequence annotations.
**Table S6**	Genotype-group statistics, additive SNP effects, and LG13 population-structure and genotype-quality sensitivity analyses.
**Table S7**	Genomic context, predicted protein lengths, and functional annotations of prioritized candidate genes.
**Table S8**	Five-tissue mean TPM values and High-versus-Low DESeq2 results for candidate genes, including explicit low-expression prefilter status.
**Table S9**	WGS–Sanger genotype concordance, independent-population validation, individual RT-qPCR data, and validation primer information.
